# Programmable Multifunctional Bistable Structures for Energy Transfer and Dissipation

**DOI:** 10.1002/advs.202518883

**Published:** 2026-02-08

**Authors:** Xin Na, Jincong Zhang, Zhicheng Chen, Venkatarao Selamneni, Haotian Chen, Hadi Heidari, Morteza Amjadi

**Affiliations:** ^1^ James Watt School of Engineering University of Glasgow Glasgow UK

**Keywords:** actuator, bistable mechanism, energy dissipation, quick stimulus response, targeted delivery

## Abstract

Bistable structures exhibiting snap‐through behavior are prevalent in nature, enabling rapid transitions between two stable states upon external stimuli. Considering such a process is accompanied by a dramatic energy conversion, here, a multifunctional bistable system composed of asymmetric bistable beams with programmable motion patterns is developed. Unlike symmetric bistable beams, the asymmetric bistable structures store greater strain energy while requiring lower activation force. The energy density of the system can be tuned by adjusting geometric parameters, type of material, and the number of beams incorporated. Experiments indicate that a three‐beam system manufactured from polylactic acid projects a sphere–comparable in weight to the beams–to a height 35 times its diameter, representing a 41% increase in energy transfer efficiency compared to a single beam of identical geometry. Leveraging the programmability and high energy conversion density features of the system, we showcase its versatility in applications including targeted payload delivery, rapid stimuli‐responsive actuation, and biomedical stents. Additionally, the capability of the system to dissipate impact energy is investigated, underscoring its potential for shock absorption.

## Graphical abstract

Utilizing the energy conversion characteristics of asymmetric bistable beams, this study develops a programmable multifunctional system composed of multiple bistable beams for energy transfer and dissipation. The high energy density enables the system to demonstrate potential in transient scenarios such as target delivery and shock absorption.

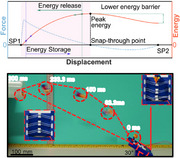

## 1 Introduction

As material science advances, a variety of smart materials are emerging on the horizon [1, 2, 3, 4, 5]. Since these materials can respond to diverse external stimuli and have a broadly adjustable range of properties [6, 7], they have greatly promoted the development of soft robots [8, 9], biomedical devices [10, 11], and other technologies [12, 13, 14]. In addition to intrinsic properties of smart materials, researchers are also seeking inspiration from ancient wisdom and nature to better harness the characteristics of smart materials. For instance, the kirigami design has been employed to increase the load capacity and stretchability of functional materials [15, 16]. Benefiting from the bistable mechanism, Venus flytrap leaves could close in 100 ms [17, 18], and beaks of hummingbirds shut swiftly when predatory [19]. Based on these natural observations, several studies have focused on the development of faster and stronger soft actuators/robots utilizing the rapid energy conversion characteristics of bistable mechanisms [20, 21, 22, 23].

A bistable structure is a typical nonlinear mechanical system possessing two local minimum potential energy positions; that is, there are two stable equilibrium states [24, 25]. When stimulated by an external stimulus, the structure rapidly realizes the switching of the stable locations, generating a snap‐through phenomenon. During the snap‐through process, the energy stored in the structure is converted into kinetic energy, leading to rapid motion and amplified force output [26, 27, 28]. Therefore, through the rational design of the bistable structure, it is possible to generate a large stroke force and release the energy with a small triggering load [29, 30, 31, 32]. Additionally, when bistable structures reach the stable states after snap‐through, they no longer require continuous energy supplies to maintain the state (i.e., they could standby for a long duration without power consumption) [28]. A case in point is the soft grippers developed by utilizing such a property that could grasp an object and hold it for a prolonged period without energy input [33, 34, 35].

Bistable structures have been studied for decades [36, 37], with a large body of research focused on theoretical investigations [38, 39, 40]. Until recent years, researchers employed bistable structures in soft actuator and robotic designs, leveraging their energy conversion features during the snap‐through process to enhance the system's autonomous motion capabilities. In 2018, T. Chen et al. reported an untethered, soft swimming robot actuated by a bistable element [29], utilizing media temperature changes to realize pre‐programmed directional propulsion. J. Sun et al. introduced a soft robot with a bistable module that could jump more than five times its body height [31]. In 2024, Q. Guo et al. presented a gas‐actuated bistable structural jumping robot achieving up to 12.7 times body‐length height jumps and up to 20 times body‐length distance jumps [23]. Despite these studies achieving fast and powerful motion of robots/actuators utilizing bistable structures, their integration designs increase the weight and volume. Furthermore, the geometric structure of the robots is constrained as the bistable modules typically feature irregular architectures, significantly limiting the functionality of robotic systems to tackle diverse and complicated tasks. A single bistable unit not only limits the magnitude of output energy but also restricts the direction of energy release. This greatly reduces the controllability of system movement, making it difficult to adjust the performance depending on varied requirements. Therefore, there remains significant research potential in how to exploit the programmed motion and energy conversion natures of bistable structures to better integrate into robotic/actuator systems to enhance their rapid and accurate deployment capabilities.

Considering the current deficiencies of bistable structures in robotics/actuators and inspired by dominoes capable of accumulating energy during the initial period and releasing it in multiple directions, this study presents a multifunctional bistable energy storage and transfer (BEST) system comprising an array of programmed bistable beams with asymmetric equilibrium states. We study the kinematic characteristics of bistable beams with different geometrical parameters through finite element analysis (FEA) and experiments, confirming that the V‐shaped asymmetric bistable structure features a lower energy barrier and higher energy output when snap‐through from one stable state to another (the ratio of transferred energy to the required input energy can be up to 16). Subsequently, energy conversion behaviors of the BEST system composed of bistable beams designed in serial or parallel configurations are investigated. Particularly, a series‐connected system manufactured from polylactic acid (PLA) accumulates energy sequentially and releases it at the terminal in a very short time (up to 14 mJ released in 30 ms) after being triggered, propelling a sphere (10 mm in diameter and 4 grams in weight) to an initial velocity of approximately $2650\;\text{mm}\;s^{-1}$ and a flight altitude of 35 times its diameter. Compared to recent studies on achieving autonomous motion in robotics/actuators using bistable mechanisms (see Table S1, Supporting Information), our work provides outstanding energy release capability. Furthermore, we demonstrate the programmability of the BEST system for various energy transfer applications ranging from precise delivery of robots (including vector projecting, obstacle vaulting, and targeted ingress), launching small‐scale gliders, and deploying it as a vascular stent. Finally, the energy dissipation capability of the BEST system is characterized through weight free‐falling tests, showing effective shock absorption by reducing the peak acceleration from 90.8 to 36.9 g (g is the acceleration of gravity). To our knowledge, this is among the initial attempts to date that achieves energy transfer and accumulation by utilizing a multi‐level asymmetric bistable beam structure.

## 2 Results and Discussion

**FIGURE 1:**
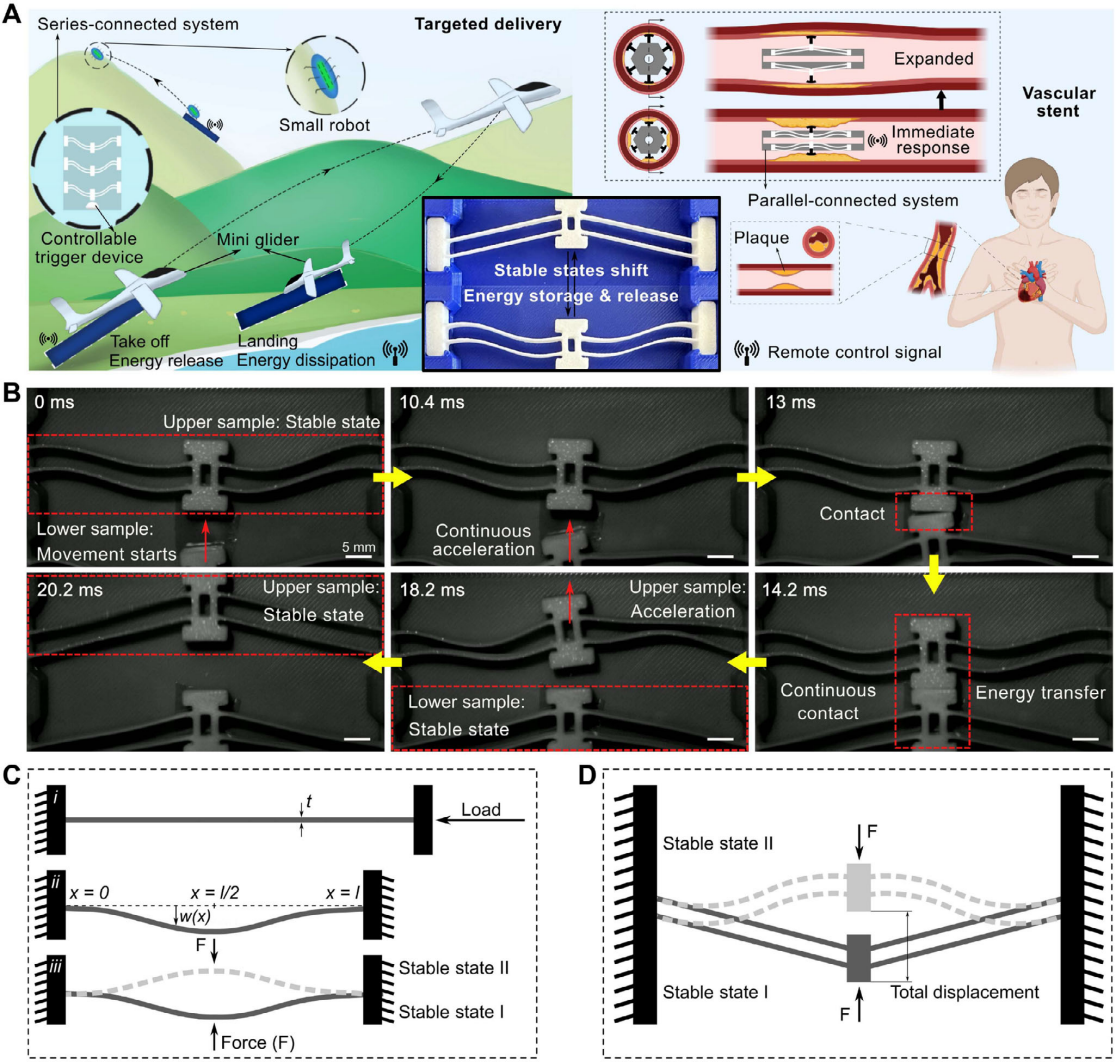
Concept of the system and the principle of bistable beams. A) Schematic diagram of the BEST system and the applications, partly created with BioRender.com, with permission. B) Slow motion of the movement and energy transfer process between two bistable beams. C) Snap‐through of a DCBB under a center point force. D) Snap‐through of VSBBs under center point force. Scale bar, 5 mm.

Figure 1A schematically illustrates the working principle and potential applications of the BEST system. In contrast to most previous studies that focused only on harnessing the energy release process of a single bistable structure during the snap‐through, the multi‐beam structure with a programmed motion pattern can accumulate or absorb more energy, leading to a greater energy release or dissipation. A series‐connected BEST system (left panel in Figure 1A) enables efficient energy accumulation and release at the terminal, which could be employed as a remote dispatch device for robots or gliders. On the other hand, a parallel‐connected BEST system (right panel in Figure 1A) can be activated to simultaneously transfer energy in multiple directions. For example, the system can be implemented as an implantable vascular stent to tackle coronary artery disease. In addition to the energy release, the BEST system has energy dissipation properties in the opposite displacement direction, making it highly promising in the field of shock absorption. To more vividly illustrate the motion behavior of a series‐connected BEST system, a high‐speed camera was utilized to record the movement pattern and interaction of two snapped beams (same geometry). As shown in Figure 1B, the lower structure is triggered to snap‐through, and the strain energy stored in the buckled beams is continuously converted into kinetic energy, accelerating the central slider. Subsequently, the lower sample impacts the upper sample, activating it to snap through. Since the upper sample accumulates the energy from the lower one in the impact, it obtains more kinetic energy (visualized as snap‐through in a shorter time).

### 2.1 Design Rationale and Characterization of Bistable Beams

**FIGURE 2:**
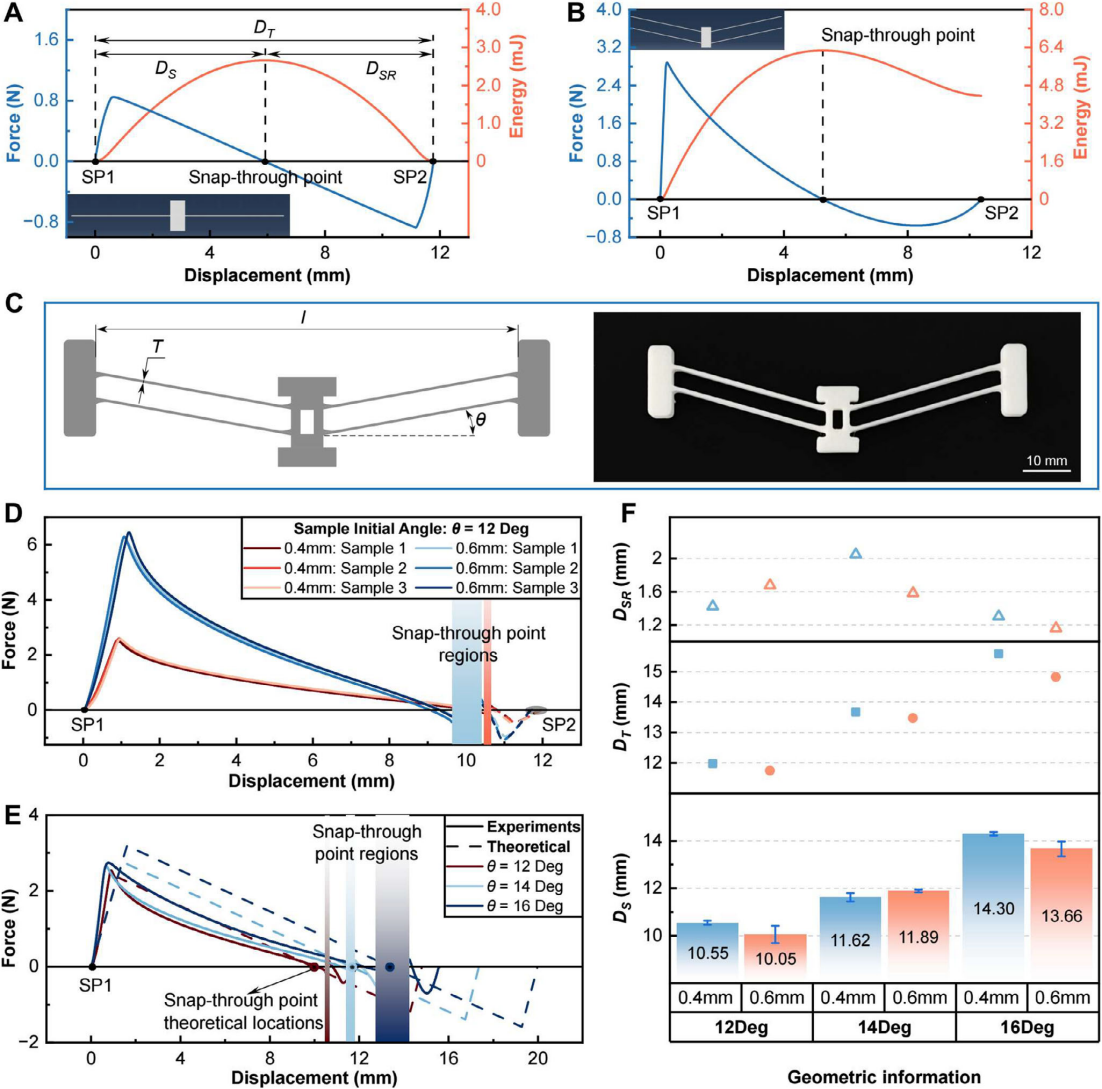
Motion characteristics and design optimization of bistable structures. A) Simulation results of the force‐displacement curve and energy landscape of the DCBB. The inset image is the model used for FEA simulation. B) Simulation results of the force‐displacement curve and energy landscape of the VSBB. The inset image is the model used for FEA simulation. C) Geometrical design (left) and 3D‐printed model (right) of a VSBB. D) Force‐displacement test results of VSBBs with the initial inclination angle of 12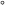 (the dashed lines indicate testing performed from SP2). Three identical samples for each geometrical condition are tested. E) Comparison of force‐displacement curves obtained from experiments and theoretical calculations. F) The average displacements from SP1 to the snap‐through point (bottom), the total displacements from SP1 to SP2 (middle), and the displacements from SP2 to the snap‐through point (top) of the bistable structures with different geometrical parameters from force‐displacement tests.

Bistable beam structures are selected in this study because of their quasi‐1D characteristics, offering several benefits including concentrated actuation forces, simple mechanical models, and broad theoretical research foundations [38, 39, 40]. Figure 1C illustrates the snap‐through of a double‐clamped single beam (DCBB), which is buckled under a compression load. The force applied at the center point drives the beam to snap‐through, creating a basic classical symmetric bistable structure. An asymmetric V‐shaped bistable beam (VSBB) structure can be obtained by introducing an initial inclination angle instead of compression, as shown in Figure 1D. To facilitate subsequent clarification, we define the displacement from stable point I (SP1) to the snap‐through point as $D_{S}$, the displacement from stable point II (SP2) to the snap‐through point as $D_{SR}$, and the total displacement from SP1 to SP2 as $D_{T}$ (see Figure 2A). To compare the properties of DCBB and VSBB, we simulated their motion pattern using FEA software ABAQUS (see Supporting Text 1 and Figure S1, Supporting Information). As Figure 2A demonstrates, the snap‐through process of a DCBB is symmetric, meaning an identical critical buckling load $F_{\mathrm{cr}}$  is required to snap‐through from different stable states. In contrast, for a VSBB, the amount of $F_{\mathrm{cr}}$  required to snap‐through from SP1 to SP2 is considerably greater than that required to snap‐through from SP2 to SP1 (Figure 2B). Consequently, more energy is stored in the beam when moving from SP1 to the snap‐through point. Moreover, the snap‐through process from SP2 to SP1 requires only a smaller triggering force (i.e., a lower energy barrier) but results in a more dramatic energy release. These results confirm that VSBBs are more appropriate for applications where a violent energy release is required under a small stimulus. Therefore, we chose VSBB as our basic model for further characterization and analysis.

Figure 2C illustrates the structural design and the key parameters of a VSBB, where *l* is the span of the snap‐through region, *T* is the thickness of the beams on each side, and θ is the initial inclination angle. For the simplicity of fabrication and rapid proof‐of‐concept, fused deposition modeling (FDM) 3D printing of PLA was chosen for sample manufacturing, as shown in Figure 2C. We fixed the dimension of *l* at 70 mm as well as the construction height to 6 mm. To enhance the lifetime of the structure, fillets were added to the beam joints to prevent them from fracturing. We designed joint units on both sides for fast assembly and replacement of VSBBs on designed frames (see Figure S2 for details, Supporting Information). The frames for clamping the samples were also fabricated by FDM 3D printing with PLA.

To characterize the motion patterns and snap‐through processes of VSBBs, their force‐displacement curves were measured by a motorized force tester (see the Experimental Section for details). Samples with different initial inclination angles from 12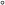 to 20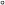 (a sample of every two degrees) were manufactured and tested. Figure 2D illustrates the force‐displacement curves for VSBBs with a constant θ of 12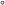 and thicknesses of 0.4 and 0.6 mm. It can be observed that the snap‐through point is determined by the initial inclination angle, while the thickness of beams greatly influences the magnitudes of $F_{\mathrm{cr}}$  and $-F_{\mathrm{cr}}$  (see Figure S3 for the results with various θ, Supporting Information). The snap‐through point approaching SP2 enables VSBB to store more energy in the $D_{S}$ region. Meanwhile, the energy barrier that requires overcoming to trigger VSBB snap‐through from SP2 is lower. However, when θ exceeds 18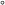, the force is always higher than zero during the motion of the VSBBs from SP1 to SP2 (see Figure S4, Supporting Information), meaning that the samples are unable to snap‐through and the structures lose their bistability.

Considering the bistable behavior is a buckling instability phenomenon, it is sensitive to the initial geometric imperfections [41]. Moreover, the 3D printing process is prone to manufacturing imperfections given the scale of our VSBB design. Therefore, to ensure the validity of subsequent experimental results, we characterized potential geometric imperfections in the 3D‐printed specimens (18 samples with θ = 14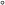 and *T* = 0.4 mm) and evaluated the impact of these imperfections through force‐displacement curve measurements. The measured beam thickness is 0.453 mm ± 0.011 mm, about 13% higher than the design dimension. Scanning electron microscope (SEM) images of the beam cross‐section reveal acceptable print quality but the widespread presence of various imperfections (see Figure S5, Supporting Information). However, from a macro perspective, the measurement results of the force‐displacement curve present adequate repeatability (see Figure S6, Supporting Information), with the coefficient of variation for $F_{\mathrm{cr}}$  being 4.17%, and the coefficient of variation for the location of the snap‐through point being only 1.62%. It indicates that the manufacturing imperfections caused by our printing setup do not have a considerable influence on the buckling behavior of VSBBs.

To further understand the kinetic properties of VSBBs, a theoretical modal analysis was conducted. The buckling mode of DCBBs can be derived according to the following differential equation for beam‐columns [42]:

(1) $$EI\frac{d^{4}w}{dx^{4}}+p\frac{d^{2}w}{dx^{2}}=0$$

The equilibrium equation describes the axial load on the beam when the transverse load equals zero. *w = w(x)* represents the lateral displacement of the beam, and *E* and *I* are the Young's modulus and moment of inertia of the beam, respectively. Since the beam is double‐clamped, it satisfies the boundary conditions at the fixed ends $w\left(0\right)=w^{\prime }\left(0\right)=w\left(l\right)=w^{\prime }\left(l\right)=0$. By solving this homogeneous Sturm‐Liouville problem (see Supporting Text 2, Supporting Information), the beam buckling modes of a DCBB can be described (see Figure S7, Supporting Information). Based on the above mathematical derivations and an analytical theory previously reported by J. Qiu et al. [39], the following key conditions can be obtained to describe the force‐displacement curve of a bistable beam with an initial stable state of the first buckling mode (i.e., *i* = 1):

(2) $$\left\{\begin{array}{cccc} F_{\text{cr}} & \approx 740\frac{EIw_{1}\left(l/2\right)}{l^{3}},\; & -F_{\text{cr}} & \approx 370\frac{EIw_{1}\left(l/2\right)}{l^{3}}\\ D_{\text{cr}} & \approx 0.16w_{1}\left(l/2\right),\; & D_{\text{-cr}} & \approx 1.92w_{1}\left(l/2\right)\\ D_{S} & \approx 1.33w_{1}\left(l/2\right),\; & D_{T} & \approx 1.99w_{1}\left(l/2\right) \end{array}\right.$$

VSBBs satisfy the snap‐through conditions outlined by J. Qiu et al., specifically, the ratio of $w_{1}\left(l/2\right)$ to the thickness of the beam is greater than 6, and the second mode (*i* = 2) is constrained [39]. Also, when θ is small, the VSBBs and the first buckling mode curved beam (*i* = 1) are geometrically identical. In fact, when the VSBB is at SP1, a tiny displacement applied is sufficient to switch the structure into the first mode. Thus, the snap‐through process of VSBBs can be approximately depicted by Equation (2). The detailed calculation procedure is available in the Supporting Information (see Supporting Text 3).

To validate the theoretical formulation, the force‐displacement curves obtained from theory are compared with measurements in Figure 2E. When the thickness and construction height of VSBBs are fixed (i.e., fixed moment of inertia I), the required critical buckling loads $F_{\mathrm{cr}}$  and $-F_{\mathrm{cr}}$  are positively correlated with the initial inclination angle because $w_{1}\left(l/2\right)=\left(l/2\right)tan\theta$. Furthermore, the snap‐through point is only determined by $w_{1}\left(l/2\right)$, which is consistent with the conclusion drawn from Figure 2D. It is noted that the experimental displacements $D_{-cr}$ and $D_{T}$ appear earlier compared to the theoretical values. This can be explained by the V‐shaped structure of VSBBs itself and the presence of fillets at the joints, which effectively suppress the transition phase of the structure from the third buckling mode (*i* = 3) to the first buckling mode (*i* = 1). The reduction in $D_{T}$ while $D_{S}$ remaining the same refers to a decrease in the traveling distance $D_{SR}$ to trigger the VSBB to snap‐through from SP2 to SP1, which is beneficial to facilitate the VSBB triggering. Figure 2F shows the results of the displacement $D_{S}$ for a variety of VSBBs with θ of 12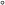, 14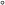, and 16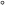, the total displacement $D_{T}$, and the displacement $D_{SR}$ for these angles. It can be observed that the values of $D_{S}$ and $D_{T}$ both increase with increasing θ, while the value of $D_{SR}$ remains constant at around 1.6 mm independent of θ. Also, VSBBs with the same θ but different thicknesses exhibit excellent uniformity.

The universality of VSBB's parametric structure under diverse dimensional scales and materials was further explored. We employed the same fabrication method to print the VSBB models with three different materials (i.e., acrylonitrile butadiene styrene (ABS), and thermoplastic polyurethane (TPU)) at different scales (the model with θ = 14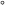 and *T* = 0.6 mm scaled proportionally up and down). By measuring their $F_{\mathrm{cr}}$  and comparing them with the theoretical values in Equation (2) (see Supporting Text 4 and Figure S8, Supporting Information), we proved that the dynamic behavior of the VSBBs is predictable and controllable across different scales and materials. With these basic kinetic parameters, we further investigated the energy output and layout design of the BEST system with multiple VSBBs incorporated.

### 2.2 Series‐Connected BEST System: Design, Optimization, and Performance Evaluation

**FIGURE 3:**
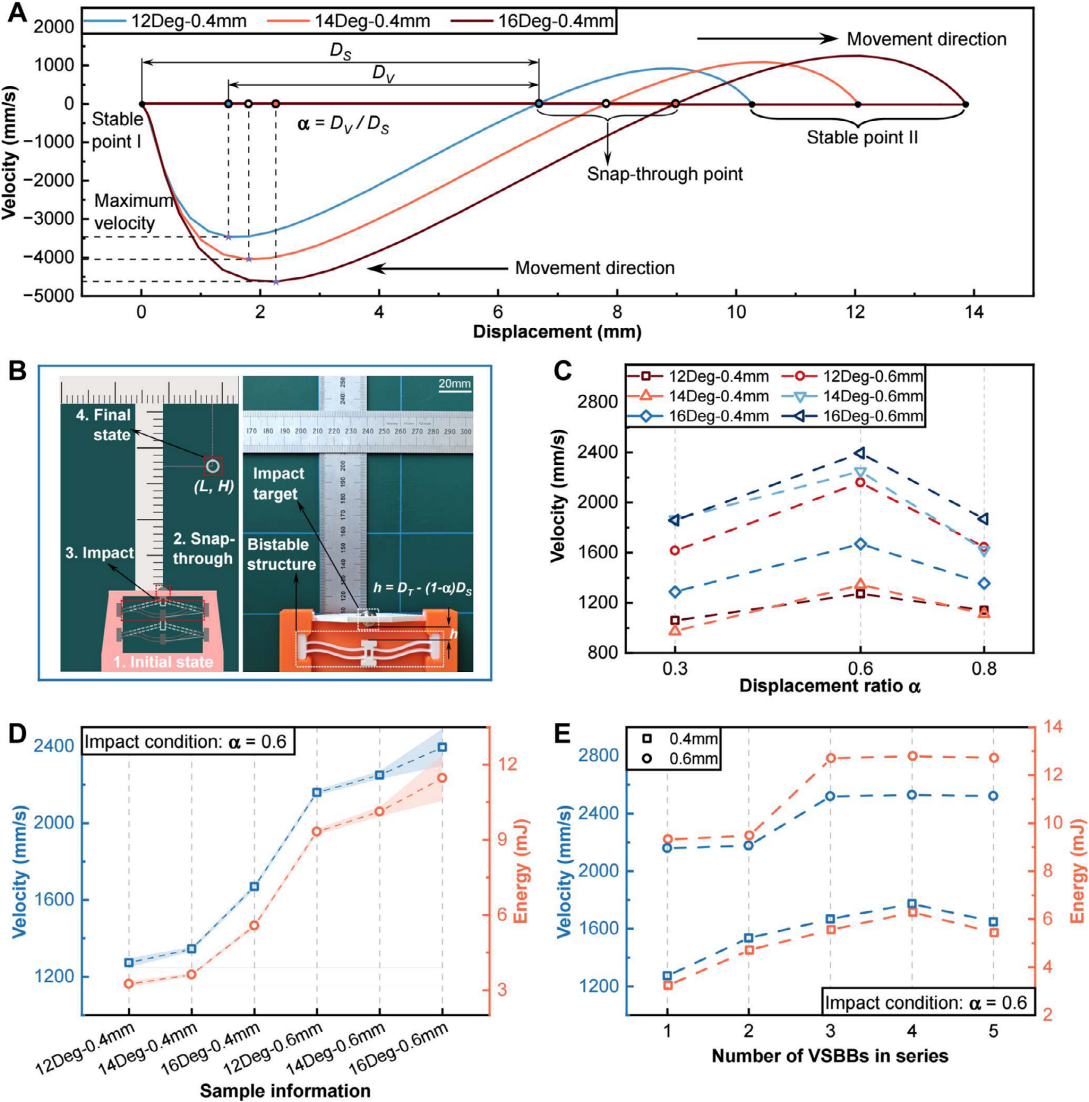
Energy accumulation and transfer performance of series‐connected VSBBs. A) Simulation results of velocity distribution of the VSBBs during snap‐through between two stable points. B) Illustration (left) and the photo (right) of the experimental system setup of sphere impact tests. C) Results of initial velocity received by the sphere when individual samples with varying geometrical parameters impacted the sphere at different positions. D) Results of the initial velocity and kinetic energy received by the sphere when individual samples with different geometrical parameters impacted the sphere at α = 0.6 position. E) Results of the initial velocity and kinetic energy received by the sphere when samples with different quantities and thicknesses of initial inclination angle of 12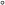 impacted the sphere at α = 0.6 position.

Before building the BEST system with programmed beams, FEA simulations were initially conducted to obtain the distribution of velocity at the central slider of VSBBs with different initial inclination angle θ (see Supporting Text 5 and Figure S9, Supporting Information). As presented in Figure 3A, the absolute maximum velocity of the central slider occurs at a location close to SP1 and increases when θ value is increased. By defining $D_{V}$ as the displacement from the snap‐through point to an arbitrary point in the SP1 interval, we obtained the displacement ratio as $\alpha =D_{V}/D_{S}$. The simulation results revealed that the maximum velocity of the central slider is reached at approximately α = 0.6. Benefiting from the detailed displacement data of VSBBs with different geometric parameters in Figure 2F, the value of α can be accurately controlled to find the position where the maximum velocity of VSBBs occurs.

The energy output of VSBBs during the motion was evaluated by a sphere impact experiment, as depicted in Figure 3B. A 3D‐printed holder was fabricated to position a metal sphere (10 mm in diameter and 4 grams in weight) on top of a VSBB. When the beam is triggered to snap‐through, the stored energy is released and transferred to the sphere after being impacted. The initial velocity (kinetic energy) obtained due to the impact event was next calculated by measuring the end position, i.e., the final lateral traveling length *L* and vertical elevation *H* of the sphere, as shown in Figure 3B (see Supporting Text 6 for detailed solution steps, Supporting Information). By adjusting the distance $h=D_{T}-\left(1-\alpha \right)D_{S}$ between the sphere and the VSBBs, we controlled the value of α when the impact occurred.

Figure 3C presents the average initial velocity of the sphere obtained from the impacts. Individual VSBBs with different geometric parameters were tested by changing α value to 0.3, 0.6, and 0.8. As depicted, the sphere reaches the maximum initial velocity under the condition of α = 0.6 for all VSBBs tested. The magnitude of the initial velocity is positively correlated with both the initial inclination angle and thickness of VSBBs. The initial velocity and kinetic energy under the condition of α = 0.6 are plotted in Figure 3D. The samples with θ = 16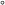 and *T* = 0.6 mm endowed the sphere with an average initial velocity of around $2400\;\text{mm}\;s^{-1}$, which translates into an average initial kinetic energy of approximately 11.5 mJ. It is 210% and 80% greater than the kinetic energy of those VSBBs with θ = 12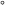 & *T* = 0.4 mm and θ = 16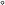 & *T* = 0.4 mm, respectively. Additionally, by processing the force‐displacement results for VSBBs, we obtained the average energy input required to activate VSBBs with different geometric parameters to snap‐through (see Table S2, Supporting Information). The results reveal that the ratio of transferred energy to the required input energy reaches to 16.

After assessing the energy release of the individual VSBB structures, the performance of BEST systems comprised of multiple VSBBs positioned in series was further investigated (see Figure S2 for the detailed arrangement of the system, Supporting Information). Figure 3E demonstrates the average initial velocity and initial kinetic energy endowed to the sphere by up to five VSBBs with θ = 12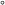. The frame structures were manufactured to align the VSBB so that the first triggered sample impacts the subsequent one when its central slider moves to α = 0.6 position to guarantee the efficiency of the energy transfer. The impact events were then serially programmed across all VSBBs in the BEST system at the optimized position. According to Figure 3E, the energy accumulation performance of the system is enhanced by increasing the number of VSBBs and gradually saturated at three series VSBBs. This trend can be explained by the considerable energy dissipation through the impact events; thus adding more beams in series does not increase the energy infinitely. Nevertheless, compared to the individual VSBB, the system composed of three 0.4 mm thick beams produces 71% more initial kinetic energy received by the spheres.

To identify the optimal performance of the systems made of three series beams, different combinations of VSBBs with a fixed thickness of 0.6 mm and varied θ were tested. The results of the average initial velocity, initial kinetic energy, and vertical elevation of the sphere are provided in Table 1. Different from individual VSBBs (i.e., the larger θ is, the more energy can be released), the group of three θ = 16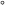 samples provided the lowest energy transfer. For a larger θ, $-F_{\mathrm{cr}}$  required to trigger the snap‐through is bigger, hence the efficiency of energy accumulation between VSBBs decreases as more energy is needed to activate series beams. Moreover, samples with larger θ have more intense buckling during the snap‐through process, making them prone to plastic deformation (see Figure S10, Supporting Information), which in turn can further deteriorate the system energy release. The highest energy accumulation capacity among the four tested groups was achieved by the combination of three θ = 14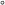 samples, giving the sphere an initial velocity of around $2650\;\text{mm}\;s^{-1}$ and elevating it more than 35 times its diameter in the vertical direction. Moreover, this configuration possesses the energy transfer ratio of over 22, around 40% greater than that of a single VSBB.

**TABLE 1:** Results of the sphere impact tests for different compositions of three‐VSBB system.

Composition	3 * 12 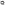	3 * 14 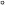	3 * 16 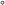	12 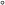 ‐14 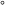 ‐16 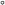
**Initial velocity (mm/s)**	2519.8	2655.9	2132.7	2448.3
**Initial kinetic energy** **(mJ)**	12.70	14.11	9.10	11.99
**Vertical elevation (mm)**	324	359	225	304

Additionally, the energy release of the series‐connected BEST system is unidirectional. When VSBBs are combined in a parallel configuration, as shown in Figure 1A (right panel), the energy is released in the programmed directions. In this case, the energy release performance in each direction depends on the geometric parameters of VSBBs. Therefore, the energy release characterization of individual VSBBs in Figure 3C is also applicable to the design of the parallel BEST systems.

### 2.3 The BEST System for Energy Transfer

We have captured the kinematic characteristics, the energy release capability, and the energy transfer and accumulation of the VSBBs. In this section, we focus on promising applications that harness the energy release and transfer properties of the BEST system. We also investigate approaches for automatic triggering of the BEST system according to different configurations and operating circumstances.

#### 2.3.1 Application of Series‐Connected BEST System

VSBBs connected in series can significantly increase the energy released, making them appropriate for deployment in applications requiring rapid energy accumulation/release. For automatic triggering of the system, an appropriate input is necessary as the trigger generator. The essential requirements for the trigger source are generating the displacement $D_{SR}$ and overcoming $-F_{\mathrm{cr}}$ , driving the structure to snap‐through.

**FIGURE 4:**
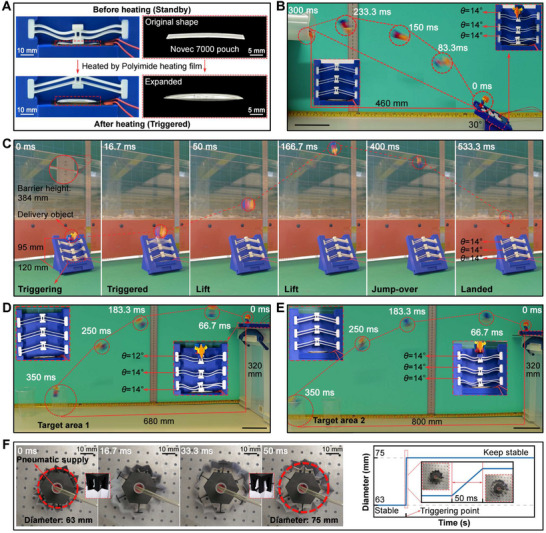
The BEST system for energy transfer applications. A) The principle of the system triggering utilizing a Novec 7000 liquid pouch. B) Result of vector projecting of a small robot (VSBB configuration: 14
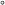
‐14
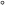
‐14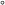). C) Result of assisting a small‐scale robot overpassing an obstacle (VSBB configuration: 14
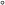
‐14
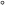
‐14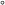). D) Result of accurate targeted delivery of a small robot (VSBB configuration: 14
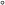
‐14
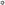
‐12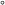). E) Result of accurate targeted delivery of a small robot (VSBB configuration: 14
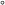
‐14
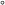
‐14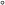). F) The verification of the concept of the parallel‐connected BEST system as a vascular stent. The inset images represent the stable‐state transitions of VSBBs before and after BEST system activation. Unlabeled scale bar, 100 mm.

As a suitable option, the low‐boiling‐point engineered fluid Novec 7000 (boiling point of 34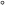 C at atmospheric pressure) satisfies the above requirements since it evaporates and expands quickly in a sealed space, producing sufficient deformation and force to trigger the system. Novec 7000 has been widely exploited by researchers in recent years. Examples include liquid pouch motors powered by Novec 7000 [43, 44, 45, 46, 47], and a flexible light and heat seeking robot [48]. One of the most popular methods of encapsulating Novec 7000 liquid pouches is the hot press process [43, 44, 45, 47, 49]. Here, we utilized a similar encapsulation method to fabricate the liquid pouches (see Supporting Text 7 and Figure S11, Supporting Information). By characterizing different sizes of the liquid pouches (see Supporting Text 8 and Figure S12, Supporting Information), the pouch with a dimension of 32 mm × 7 mm was finally selected as the trigger source for the series‐connected system. The selected Novec 7000 pouch was expanded by using a polyimide (PI) heating film as the heating source, as shown in Figure 4A.

As proof‐of‐concept demonstrations, series‐connected BEST systems have been designed to perform different tasks. In the first example, we employed the system for realizing vector projecting of small robots or critical cargoes (a Lego Minifigure of 43 mm in height and 4.44 grams in weight was used as a model sample, see Figure S13, Supporting Information). As shown in Figure 4B, a BEST system consisting of three VSBBs with θ = 14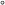 and *T* = 0.6 mm, the Novec 7000 liquid pouch, as well as a PI heating film was placed with a block (30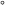). The system enabled the object enter a 60 mm diameter pipe, which was 460 mm away at an upper tangent angle of about 30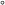 (see Supporting Text 9 and Movie S1, Supporting Information). Subsequently, obstacle vaulting of small robots was conducted, as depicted in Figure 4C. The BEST system consisted of three VSBBs with θ = 14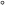 and *T* = 0.6 mm, and placed together with a 75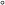 block. Then the system was actuated approximately 20 seconds after heating, enabling the object jump over the fence (height 384 mm) to the destination after around 530 ms (see Movie S2, Supporting Information).

To illustrate the programmability, series‐connected BEST systems composed of VSBBs with varying initial inclination angle θ were constructed for targeted delivery. Figures 4D,E present the system accomplishing two tasks of precision ingress of small robots or critical cargoes over different distances. For the task in Figure 4D, VSBBs with θ of 14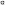, 14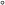 and 12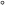 (all with 0.6 mm thickness) were incorporated together to successfully transport the object to a 65 mm diameter container located 680 mm away (see Movie S3, Supporting Information). As the system required more energy release to accomplish targeted delivery for a higher distance in Figure 4E, the VSBB of θ = 12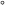 was replaced with the one with θ = 14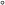. As a result, the BEST system sent the object to the same container yet at 800 mm away (see Movie S4, Supporting Information).

Lastly, the potential of our BEST system to serve as a programmable small‐scale aircraft launcher was presented. We handcrafted a glider model with a wingspan of 368 mm and 5.07 grams of weight (see Figure S13, Supporting Information). A system with θ of 14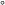, 12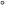 and 12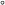 of the VSBBs (all with 0.6 mm thickness) was placed with the glider model together at 720 mm height, making the model glide for about 1150 mm (see Supporting Text 9, Figure S14, and Movie S5, Supporting Information). By programming the θ combinations of VSBBs in the BEST system, gliding distances of the glider model were tuned, reaching to around 1220 mm (for θ = 14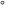, 14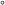 and 12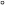) and 1300 mm (for θ = 14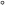, 14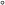 and 14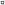) (see Movies S6 and S7, Supporting Information). Such application is highly beneficial for short take‐offs of fixed‐wing vehicles.

The above demonstrations prove that programmable energy output can be realized through an elaborately designed combination of VSBBs with different geometric parameters, delivering the object to the targeted area based on requirements. Second, programmable vectorized energy output can be achieved by modifying the angle of the BEST system placement, thereby significantly improving the system's compatibility with complex scenarios. Additionally, to prove the feasibility of the BEST system in higher‐energy‐requirement situations, we prepared a titanium alloy VSBB (VSBB model with θ = 14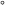 and *T* = 0.6 mm, scaled up by 1.25 times, mass: 17.1 g) for impact testing (see Experimental Section and Figure S15 in Supporting Information). It enabled a 100 g weight to obtain a flight height of roughly 178 mm, indicating that the weight gained at least 170 mJ of energy, verifying the potential of the BEST system for practical applications.

#### 2.3.2 Application of Parallel‐Connected BEST System

The system connected in series allows the accumulation and release of energy in an extremely short time and unique direction. When the VSBBs are connected in parallel, on the other hand, the direction of energy output is dependent on the orientation of VSBBs. In this situation, the energy can be distributed and transferred in multiple directions.

To illustrate the potential of parallel‐connected systems, we developed a smart stent capable of fast expansion. Coronary artery disease is caused by the buildup of fat and cholesterol in the arteries of the heart (known as plaque). The plaque may completely block blood flow over time, eventually leading to a heart attack [50]. Currently, stent implantation is an effective treatment for narrowed arteries. A metal stent with a folded balloon is delivered to the site of the vascular blockage, and the balloon is then inflated to expand the stent and push the plaque. Considering the multidirectional energy transfer property of the parallel‐connected BEST system, the unfolding of a vascular stent has been simulated. As shown in Figure 4F, six VSBBs were connected in parallel through a hexagonal frame, all of which were initially at SP2. A variable diameter ring was obtained by folding paper for simulating an elastic blood vessel. The system was actuated by a balloon that connected to a pump. Before triggering the system, the ring diameter was measured at 63 mm, while it increased to 75 mm around 50 ms after triggering, indicating the possibility of the BEST system as a smart vascular stent (see Movie S8, Supporting Information). In addition, the structure of vascular stents based on a BEST system activated by a contactless method could eliminate the need for a pneumatic module as in conventional stents. For this purpose, we demonstrated activation of a VSBB and BEST systems using contactless magnetic effect (see Movie S9, Supporting Information).

### 2.4 The BEST System for Energy Dissipation

**FIGURE 5:**
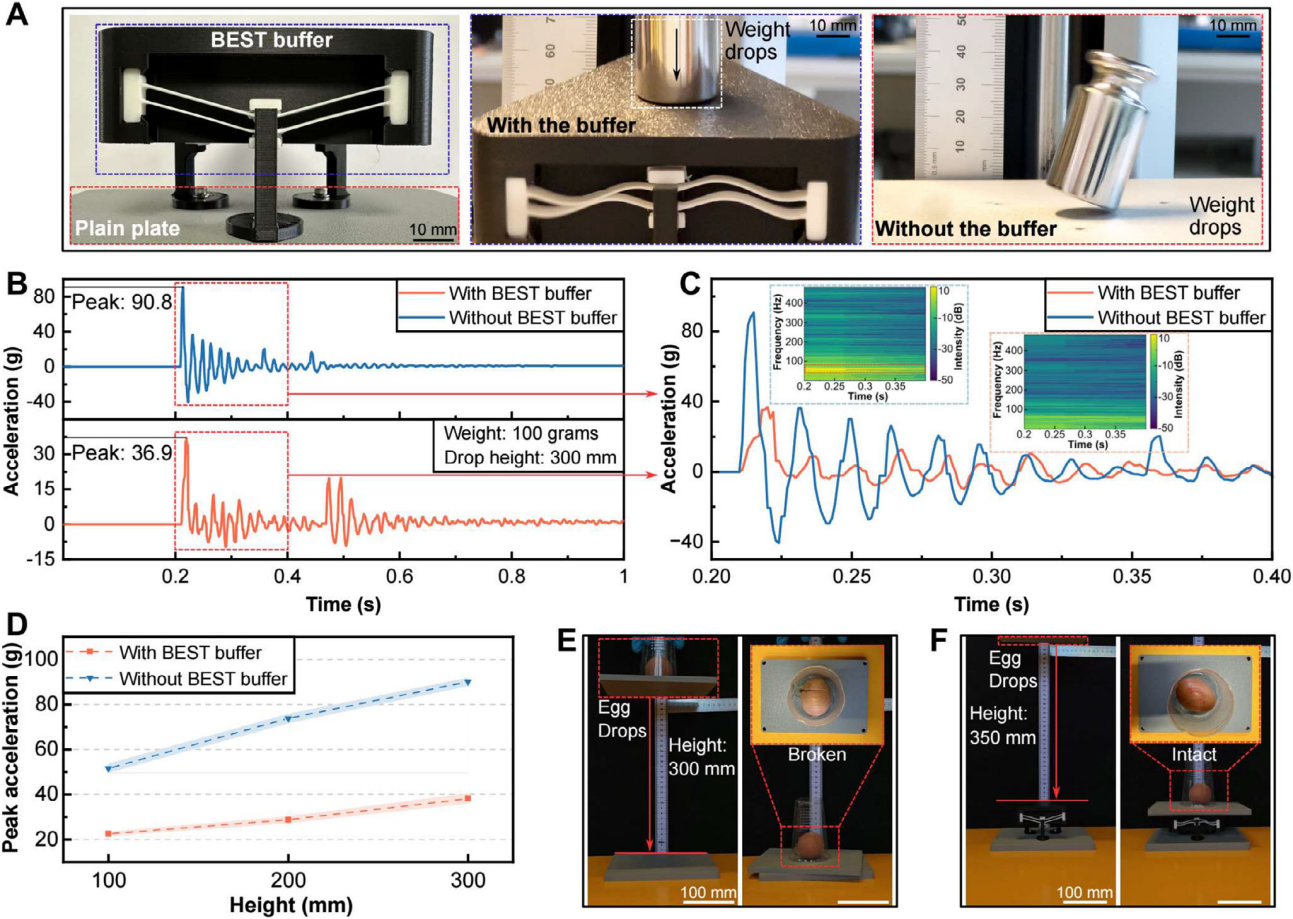
Studies on the energy dissipation potential of the BEST system. A) The BEST buffer structure diagram and the principle of weight dropping tests. B) The results of the acceleration response for the weight dropped from 300 mm. C) The local zoom‐in results of the acceleration response and spectrograms for the weight dropped from 300 mm. D) The peak acceleration results at different heights for the two test conditions. E) Raw egg falling protection test without the buffer. F) Raw egg falling protection test with the buffer.

In previous sections, we focused on VSBB configurations that achieve dramatic energy release from SP2 to SP1 by overcoming lower energy barriers (see Figure S16, Supporting Information). When a VSBB is driven in the opposite direction (i.e., from SP1 to SP2), it can absorb the energy through the buckling process. To explore such an energy dissipation property, a BEST buffer as shown in Figure 5A was designed, in which three parallel VSBBs with θ = 14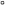 and *T* = 0.6 mm were mounted on a frame with an equilateral triangle cross‐section. A calibration weight (100 grams) was released from different heights to the surface of the buffer and a plain plate for comparison. The real‐time forces were measured by the motorized force tester to acquire the impact response under the two conditions (see Supporting Text 10 and Figure S17, Supporting Information).

Figure 5B displays the results of the acceleration response for the weight with a free‐fall height of 300 mm. It can be noticed that the peak impact acceleration drastically decreased from 90.8 g for the plain plate to 36.9 g with the introduction of the BEST buffer. To compare the difference of impact modes under the two test conditions with more details, the acceleration response data at the initial impact phase of 0.2–0.4 s is depicted in Figure 5C. By performing a short‐time Fourier transform, we obtained the spectrograms of the impact energy shown in the insert of Figure 5C. When the impact occurred directly on the plain plate, the energy was rapidly concentrated and dissipated slowly. On the contrary, in the presence of the buffer, the system absorbed most of the energy in the impact through the buckling of the VSBBs, resulting in not only a lower level of concentrated energy but also a quicker dissipation. It indicated that the buffer significantly improves the damping behavior of the system, which makes it an efficient shock absorption solution. The peak accelerations acquired at different heights are illustrated in Figure 5D. In general, the peak acceleration increased linearly with the height of the weight drops in both conditions, but the presence of the buffer reduced the peak acceleration by approximately 60 %.

To visually display the performance of the BEST buffer in practical shock absorption applications, we designed a demonstration of raw egg falling protection (see Movie S10, Supporting Information). In Figure 5E, a raw egg was released from a height of 300 mm to a plain plate, resulting in the egg breaking due to the impact. Under an identical test, another raw egg was released from a height of 350 mm to the BEST buffer surface. By dissipating the impact energy through the buffer, the egg remained intact and was effectively protected (Figure 5F). The above experiments reveal that the VSBB‐based buffer can effectively suppress impacts and enhance the damping properties, which helps to reduce the injuries caused by random impacts.

## 3 Conclusion

In this study, we developed a multifunctional energy storage and transfer system based on VSBBs, presenting high energy storage and release capacities. The system achieved a tunable energy release performance by varying the design parameters of the VSBBs. Experiments showed that for individual VSBBs (PLA), the ratio of energy transferred after snap‐through to the required triggering energy is around 16. We identified the optimal geometrical configuration to maximize the energy transfer through successive snap‐through of the series‐connected system. The sphere impact experiments indicated that the BEST system consisting of three VSBBs manufactured from PLA provides the sphere with a maximum initial kinetic energy of 14 mJ (22 times to the required input energy), which was around a 40% improvement compared to the results of an individual VSBB, enabling the sphere to obtain an initial velocity of approximately $2650\;\text{mm}\;s^{-1}$ and a vertical elevation of 35 times its diameter. Subsequently, the versatility of the system for energy transfer was demonstrated through successful targeted delivery, small‐scale glider launch, and concept validation of vascular stent. Furthermore, utilizing the energy storage property of VSBBs makes them an efficient buffer for shock absorption. The energy storage characteristic of the system can effectively reduce the peak acceleration (from 90.8 to 36.9 g) caused by impact and increase the damping.

By investigating the VSBBs and exploring different configurations of the BEST system, our study reveals that the energy storage and release performance can be programmed for different requirements, offering new avenues for the perspective of the classical bistable mechanism. Future work may explore alternative durable materials, advanced manufacturing techniques, and more controllable triggering mechanisms. Additionally, the system presents a broad range of possibilities in various fields. At smaller dimensions, the system can be implemented in biomedical fields such as drug delivery, vascular stents, and energy harvesting. Further miniaturized to microelectromechanical systems, the VSBBs can be engineered as logic gates or valves. At larger scales, the structures can be introduced to industries and space, since no continuous energy input is needed to maintain the stable states.

## 4 Experimental Section

### 4.1 Fabrication of the Modules

VSBBs for the main test and demonstrations, all the system frameworks, and the plain plate were 3D printed with PLA (Filaments: PLA Basic with 1.75 mm ± 0.03 mm, purchased from Bambu Lab) by a commercial printer (P1S, Bambu Lab). The VSBBs made from ABS and TPU materials are also fabricated by P1S, with consumable filaments (ABS with 1.75 mm ± 0.03 mm and TPU for AMS with 1.75 mm ± 0.05 mm) from Bambu Lab. VSBBs made from PLA were printed with a nozzle diameter of 0.4 mm, extrusion temperature of 220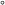 C, bed temperature of 55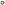 C, layer height of 0.12 mm, and wall printing speed $20\;\text{mm}\;s^{-1}$. VSBBs made from ABS and TPU adopted the same print parameter settings as PLA, modifying only the extruder temperature (260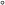 C for ABS and 230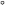 C for TPU) and bed temperature (90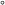 C for ABS and 35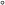 C for TPU) according to material requirements. The titanium alloy VSBB was fabricated using the selective laser sintering process (commissioned an external company, HUA‐YIXUN Technology). All printing parameters and orientation settings for PLA material property test specimens were identical to those used for printing VSBBs with PLA. The frameworks and plain plate were printed according to the default parameters of the printer. Cut‐to‐size films (metallized film, thickness 36μm, Amcor) were heat sealed by an impulse heat sealer (power: 400 W, YORKING, purchased from Amazon), and Novec 7000 (3M Company, purchased from Sigma‐Aldrich) was added quantitatively using a pipette. The glider model was handcrafted using balsa wood (purchased from Amazon).

### 4.2 Finite Element Analysis

The simulations were all conducted by the finite element software ABAQUS/CAE 2024 (Dassault Systèmes Simulia Corp.). According to ASTM D638‐14, tensile testing was performed to determine the material properties of 3D‐printed PLA in the direction of deformation (see Figure S18, Supporting Information). The results yielded a tensile modulus of 2051 MPa ± 92 MPa, a yield strength of 20.4 MPa ± 3.4 MPa, and the yield strain was 1.19%. According to ASTM D790‐17, the three‐point bending test was performed to determine the bending modulus of the 3D‐printed PLA in the direction of deformation, which was 2299 MPa ± 23 MPa (see Figure S19, Supporting Information). All data have been summarized in Table S3 (Supporting Information). The density of the material was chosen as $1240\;\text{kg}\;m^{-3}$. Considering that the VSBB primarily undergoes bending deformation during the movement process, we selected its bending modulus (2.3 GPa) for analysis, with Poisson's ratio set to 0.3. Since the kinematic characteristics of the bistable beam are consistent in the height direction, 2D VSBB models were selected for analysis in order to improve simulation speed and reduce computational complexity. The beam section of the model adopted a 4‐node plane stress element with incompatible mode (CPS4I), and the quad‐dominated method was chosen for meshing the model. Based on the trade‐off between mesh sensitivity analysis and simulation efficiency, the mesh edge length for the beam section was selected as 0.1 mm. Force/energy‐displacement simulation of bistable beams was conducted by static‐general analysis (Nlgeom = On), with displacement loads applied to actuation. Velocity‐displacement simulation of V‐shaped beams was performed by implicit dynamic analysis (Nlgeom = On), with force loads applied to actuation. The solver applied a full Newton solution technique.

### 4.3 Force and Dimension Measurements

Force measurements of all VSBBs (made from PLA, ABS, and TPU), Novec 7000 liquid pouches, and weight dropping were all performed on a motorized force tester (MultiTest 2.5‐dV, Mecmesin). The force tester platform provides multiple threaded mounting holes, so we connected and secured our samples to it through various 3D‐printed fixtures. For force‐displacement measurements of V‐shaped beams, the samples were fixed on the platform, and the force tester probe was placed in contact with the central slider of each sample moving at a velocity of $20\;\text{mm}\;\min^{-1}$ until the snap‐through occurred; then the sample was re‐mounted in the opposite direction for another measurement using the same method. The sampling frequency of the tester was set to 500 Hz when acquiring the data. Three samples were measured for each geometric parameter. For force measurements of liquid pouches, each liquid pouch and the PI heating film (size: 10 mm × 50 mm, maximum power: 1.5 W, Fangzhou Electronics) were attached together on the platform, the force tester probe was positioned in complete contact with the surface of the pouch, and the heating film was powered by a DC power supply (72‐2550, TENMA). The sampling frequency of the tester was set to 500 Hz when acquiring the data. For the weight dropping tests, four M6 threaded rods (purchased from Amazon) were used to connect the plate to the probe of the force tester. The weight from a designed height was freely dropped onto the plate/buffer. The sampling frequency of the force tester was set at 1 kHz to collect data. More details about the above experiments are provided in Supporting Texts 8 and 10. The determination of material properties for PLA specimens was performed using an Instron 5966 Tensile Testing Machine equipped with an extensometer. The beam thickness was measured using a micrometer. Three random positions were selected on each of the four beams of every VSBB, and a total of 216 data points were obtained from 18 VSBBs. The mean and standard deviation were then calculated.

### 4.4 Calculation of the Initial Velocity and Kinetic Energy of a Sphere

We set the initial position of the sphere as the coordinate origin. The coordinates (*L*, *H*) of the sphere at its highest point can be obtained through image processing. Based on the law of conservation of energy, the following relationship exists in the vertical direction (neglects all the aerodynamic drag):

$$K_{0}+U_{0}=K_{t}+U_{t}$$

 Where *K* and *U* represent the kinetic energy and gravitational potential energy of the ball, respectively. Since $U_{0}$, $U_{t}$, and $K_{t}$ are all already known, the initial velocity of the sphere can be derived. The sphere decelerates uniformly in the vertical direction (with an acceleration of ‐g), so the duration of the motion can be calculated when the initial and final velocities are known. The sphere can be regarded as moving at a constant speed in the horizontal direction, thus the velocity was calculated after determining the duration of the motion. Supporting Text 6 provides a detailed derivation of the calculation process.

### 4.5 Motion Recording and Image Processing

The slow‐motion recording in the schematic demonstration was captured with the high‐speed camera (9000 FPS, Phantom, V2511, Vision Research). SEM images of the beam cross‐section were obtained by TESCAN CLARA. The sphere impact experiment was recorded via smartphone at 4K, 60 FPS (iPhone 14 Pro, APPLE). Demonstration experiments were filmed via two smartphones in dual camera positions at 4K, 60 FPS (iPhone 14 Pro, APPLE, and Xiaomi 13 Ultra, Xiaomi). All images were taken with an iPhone 14 Pro. Images were edited by Microsoft's standard photo editor, and page layouts were completed using Inkscape. The video clips were created using CapCut and Clipchamp.

## Author Contributions

X.N. and M.A. conceived this research and designed the experiments. X.N. performed simulations and analysis. X.N. and J.C.Z. performed experiments and collected data. X.N. and Z.C.C. modeled and 3D printed the experimental samples. X.N., J.C.Z., and V.S. recorded the videos. X.N., J.C.Z., Z.C.C., and V.S. discussed and analyzed the data. X.N. conducted data visualization. M.A., H.T.C., and H.H. provided guidance and supervision. X.N. prepared the manuscript with assistance from J.C.Z., Z.C.C., and V.S. All the authors reviewed, edited, and approved the paper.

## Conflicts of Interest

The authors declare no conflicts of interest.

## Supporting information

Supplemental Movie 1

Supplemental Movie 2

Supplemental Movie 3

Supplemental Movie 4

Supplemental Movie 5

Supplemental Movie 6

Supplemental Movie 7

Supplemental Movie 8

Supplemental Movie 9

Supplemental Movie 10

Supporting Information

## Data Availability

The data that support the findings of this study are available from the corresponding author upon reasonable request.
