# Supplementary material for: Programmable Multifunctional Bistable Structures for Energy Transfer and Dissipation
Source: Adv Sci (Weinh). 2026 Feb 8;13(17):e18883. doi: 10.1002/advs.202518883 (PMC13042753; doi:10.1002/advs.202518883)
Supplement: Supplementary file 11 — Supporting Information [file ADVS-13-e18883-s011.pdf]

Supporting Information

Programmable Multifunctional Bistable Structures for Energy Transfer and Dissipation

*Xin Na, Jincong Zhang, Zhicheng Chen, Venkatarao Selamneni, Haotian Chen, Hadi Heidari, Morteza Amjadi**

James Watt School of Engineering, University of Glasgow, Glasgow G12 8QQ, UK.

*Correspondence to: Morteza Amjadi (Morteza.Amjadi@glasgow.ac.uk).

The PDF file includes:

Supplementary Text 1 to 10
Figure S1 to S19
Table S1 to S3
Legends for Movies S1 to S10
Supporting References

Other Supplementary Material for this manuscript includes the following:

Movies S1 to S10

Supplementary Texts

1. Abaqus FEA of DCBB and VSBB

The simulations were performed by the static-general analysis method (Nlgeom = On). Considering the material anisotropy introduced by 3D printing, we first measured the material properties along the primary direction of deformation in the beam:

1. According to ASTM D638-14, tensile testing was performed to determine the material properties of 3D-printed PLA in the direction of deformation (Figure S18). Valid data was obtained for five specimens. The results yielded a tensile modulus of 2051 MPa ±92 MPa, a yield strength of 20.4 MPa ±3.4 MPa, and the yield strain was 1.19%.

2. According to ASTM D790-17, the three-point bending test was performed to determine the bending modulus of the 3D-printed PLA in the direction of deformation (Figure S19). Valid data was obtained for five specimens. The obtained bending modulus was 2299 MPa ±23 MPa.

Considering that the VSBB primarily undergoes bending deformation during the movement process, we selected its bending modulus (2.3 GPa) for analysis, with Poisson's ratio set to 0.3. The density of the material is 1240 $\mathrm{kg\,m^{-3}}$.

For the simulation of the DCBB, a two-dimensional thin beam with a length of 70 mm and a width of 0.4 mm was constructed, and a slider with a length of 5 mm and a width of 10 mm was placed in the center, as shown in Figure S1A. The beam section of the model adopted a 4-node plane stress element with incompatible mode (CPS4I), and the quad-dominated method was chosen for meshing the model. Mesh sensitivity analysis indicated that when the edge length of the beam mesh is less than 0.2 mm, the impact of mesh size on simulation results can be neglected. Therefore, considering the balance between simulation accuracy and efficiency, the mesh edge length for the beam section was selected as 0.1 mm. The simulation was separated into two steps. In step 1, a displacement load of 6 mm to the central slider along the Y-axis was applied to make the beam buckle. In step 2, a displacement load was applied to the central slider, which was completely opposite to that in step 1, but both ends of the beam were clamped so that a snap-through occurred. Subsequently, the force-displacement information of the beam during the snap-through process was extracted by post-processing the data of a node on the surface of the central slider, and the energy landscape was derived by integrating the force over the displacement.

For the simulation of VSBB, a two-dimensional model was established as shown in Figure S1B. The distance between the ends of the beams is also 70 mm with a thickness of 0.4 mm connected in the center by a 10 mm $\times$ 5 mm slider. The beam section of the model adopted a 4-node plane

stress element with incompatible mode (CPS4I), and the quad-dominated method was chosen for meshing the model. And the mesh edge length for the beam section was selected as 0.1 mm. Secured the ends of the beams by adding constraints, and the structure was snap-through by the displacement load acting on the central slider in the Y-axis direction. Same as the processing of the DCBB described above, the force-displacement data of a node on the surface of the central slider was extracted, and the energy landscape was obtained through integration.

2. Modal analysis to explain the bistable behavior of the beams

For the DCBB, according to the differential equation for beam-columns,[1] the equilibrium equation describing the axial load on the beam when the transverse load equals zero is:

$$EI\frac{d^4w}{dx^4} + p\frac{d^2w}{dx^2} = 0 \tag{S1}$$

where $w = w(x)$ represents the lateral displacement of the beam, and E and I are the Young's modulus and moment of inertia of the beam, respectively. And because the two ends of the beam are clamped, the boundary conditions can be expressed as:

$$w(0) = w'(0) = w(l) = w'(l) = 0 \tag{S2}$$

The dimensionless form of the axial force is:

$$N^2 = \frac{pl^2}{EI} \tag{S3}$$

Thus, Equation (S1) can be deformed as:

$$w'''' + \frac{N^2}{l^2}w'' = 0 \tag{S4}$$

By solving the general solution of the differential Equation (S4) and applying the boundary condition Equation (S2),[2] a system of two linear homogeneous equations for constants A and B is obtained:

$$\begin{cases} A(\cos(N) - 1) + B(\sin(N) - N) = 0 \\ -A\sin(N) + B(\cos(N) - 1) = 0 \end{cases} \tag{S5}$$

Only if the system of Equations (S5) has nonzero solutions, the buckling of the beam occurs. It requires N to satisfy the following equation:

$$\sin\left(\frac{N}{2}\right)\left[\tan\left(\frac{N}{2}\right) - \frac{N}{2}\right] = 0 \tag{S6}$$

The following two equations are finally derived to describe the buckling modes of the DCBB:

Group 1: $N_i = 2\pi, 4\pi, 6\pi, ...$, $i = 1, 3, 5...$

$$w_i(x) = C_i \left[1 - \cos\left(N_i \frac{x}{l}\right)\right] \tag{S7}$$

Group 2: $N_i = 2.86\pi, 4.92\pi, 6.94\pi, ...$, *i* = 2, 4, 6...

$$w_i(x) = C_i \left[1 - 2\frac{x}{l} - \cos\left(N_i \frac{x}{l}\right) + \frac{2\sin\left(N_i \frac{x}{l}\right)}{N_i}\right] \tag{S8}$$

Where C_i are the amplitudes of the functions. With the above two groups of equations, the buckling profile of a DCBB for different numbers of modes can be accurately expressed.

3. Theoretical calculations for modal analysis of a VSBB

Based on the previous research,[3] the key parameters in the force-displacement curve of a bistable beam can be calculated by the following formulas:

$$\begin{cases} f_{\text{top}} \approx 740\dfrac{EIw_1(l/2)}{l^3}, & f_{\text{bot}} \approx 370\dfrac{EIw_1(l/2)}{l^3} \\ d_{\text{top}} \approx 0.16w_1(l/2), & d_{\text{bot}} \approx 1.92w_1(l/2) \\ d_{\text{mid}} = \dfrac{4}{3}w_1(l/2), & d_{\text{end}} \approx 1.99w_1(l/2) \end{cases} \tag{S9}$$

An example of a VSBB with an initial inclination angle of 12°and thickness of 0.4 mm is given to demonstrate the process of theoretical calculation. The parameters to be applied are shown below:

$$\begin{cases} \text{Length/Span: } l = 70\,\text{mm} \\ \text{initial inclination angle: } \theta = 12^\circ \\ \text{Beam thickness: } t = 0.4\,\text{mm} \\ \text{Beam width: } b = 6\,\text{mm} \\ \text{Young's Modulus: } E = 2300\,\text{MPa} = 2300\,\text{N}\,\text{mm}^{-2} \\ \text{Lateral displacement: } w_1(l/2) = (l/2)\tan\theta \approx 7.44\,\text{mm} \end{cases} \tag{S10}$$

For a beam with a rectangular cross-section, the moment of inertia is:

$$I = \frac{(6\,\text{mm})(0.4\,\text{mm})^3}{12} = 0.032\,\text{mm}^4 \tag{S11}$$

There are two beams in every V-shaped structure, hence the critical buckling load F_{cr} and $-F_{cr}$ need to be calculated by multiplying the original formula by 2. The following are the results of the calculation of six key parameters:

$$\begin{cases} F_{\text{cr}} = 2f_{\text{top}} \approx 1480\dfrac{EIw_1(l/2)}{l^3} = 2.36\,\text{N} \\ -F_{\text{cr}} = 2f_{\text{bot}} \approx 740\dfrac{EIw_1(l/2)}{l^3} = 1.18\,\text{N} \\ D_{\text{F}} = d_{\text{top}} \approx 0.16w_1(l/2) = 1.19\,\text{mm} \\ D_{\text{-F}} = d_{\text{bot}} \approx 1.92w_1(l/2) = 14.28\,\text{mm} \\ D_{\text{S}} = d_{\text{mid}} \approx \dfrac{4}{3}w_1(l/2) = 9.92\,\text{mm} \\ D_{\text{T}} = d_{\text{bot}} \approx 1.99w_1(l/2) = 14.81\,\text{mm} \end{cases} \tag{S12}$$

4. Bistable behavior analysis of VSBBs with different materials and scales

Based on our VSBB model with θ = 14°and T = 0.6 mm, we printed four dimensions (scaled proportionally to λ = 0.75, 1.0, 1.25, and 1.5, where λ is the scale factor) and three materials (PLA, acrylonitrile butadiene styrene (ABS), and thermoplastic polyurethane (TPU)), totaling twelve VSBB sample sets. Each sample set consisted of 5 specimens, for which the critical buckling load F_{cr} was obtained through force-displacement measurements, and then the average value $\overline{F_{cr}}$ was calculated. The $\overline{F_{cr}}$ obtained from 1.0 times scale PLA specimens were utilized as the reference value for normalizing the 12 sets of measurement results.

For the calculation of the theoretical value, substituting Equation (S11) and $w_1(l/2) = (l/2)\tan\theta$ into the first row of Equation (S12) gives:

$$F_{\mathrm{cr}} \approx 61.7 E tan\theta \frac{bt^3}{l^2} \tag{S13}$$

Since θ remains constant and all dimensions change proportionally, the theoretical value of F_{cr} can be expressed in terms of the material and dimensional scale factor as follows:

$$F_{\mathrm{cr}} \propto E\lambda^2 \tag{S14}$$

That is, it is directly proportional to the Young's modulus of the material and proportional to the square of the scale factor λ. The Young's modulus for all materials was selected as the average value from the filament data sheet (E_{PLA} = 2580 MPa, E_{ABS} = 2200 MPa, and E_{TPU} = 1190 MPa). The Young's modulus of PLA at 1.0 times scale was still chosen as the reference value for normalization. The combined experimental and theoretical results are presented in the heatmap as shown in Figure S8. The results indicated the consistency between experimental and theoretical approaches, proving that the dynamic behavior of our VSBBs is predictable and controllable across different scales and materials.

5. Velocity simulation results for the VSBB

The simulations were performed by the implicit dynamic analysis method (Nlgeom = On). The simulation object was obtained by varying the initial inclination angle of the two-dimensional model in Figure S1B. And the material parameters, boundary conditions, and meshing methods applied were also consistent with those used in Supplementary Text 1. Next, a VSBB model with an initial inclination angle of 12°and thickness of 0.4 mm is used as an example to explain the specific procedure for obtaining the velocity-displacement results. The beam section of the model adopted a 4-node plane stress element with incompatible mode (CPS4I), and the quad-dominated method was chosen for meshing the model. Mesh sensitivity analysis indicated that when the edge length of the beam mesh is less than 0.2 mm, the impact of mesh size on simulation results can be neglected. Therefore, considering the balance between simulation accuracy and efficiency, the mesh edge length for the beam section was selected as 0.1 mm.

Initially the VSBB is at stable point I, and a constant force is placed on the central slider. We fixed the load duration and adjusted the force to make the structure just capable of snap-through. The structure then halted at stable point II. We then used the same method to make the structure snap through again and back to stable point I by adjusting the magnitude of the reverse constant force. During post-processing, we extracted the velocity and displacement over time from a node on the center slider and combined the two data to generate the velocity-displacement curve shown in Figure 3A in the main text. Figure S9 shows the simulation results of the kinematic state of the VSBB model with an initial inclination angle of 12° and thickness of 0.4mm at different moments. The results also confirm that the design of the central slider effectively constrains the appearance of the second mode.

6. Calculation process for solving the initial velocity and kinetic energy of the sphere in the sphere impact experiments

The sphere flew out after being impacted, and we recorded the flight trajectory of the sphere through a camera. Subsequently, the position coordinates (L, H) of the sphere when the vertical velocity was zero were obtained through video processing (defining the sphere position at its initial state as the coordinate origin). Under the prerequisite of neglecting all the aerodynamic drag, the following relationship exists between the initial velocity V_{y0} and the final velocity V_{yT} of the sphere in the vertical direction according to the law of conservation of energy:

$$\frac{1}{2}mV_{y0}^2 + 0 = \frac{1}{2}mV_{yT}^2 + mgH \tag{S15}$$

Where m is the mass of the sphere and g is the acceleration of gravity. In our experimental scenario, $V_{yT} = 0$. By deforming Equation (S13), the initial velocity of the sphere in the vertical direction V_{y0} can be derived as:

$$V_{y0} = \sqrt{2gH} \tag{S16}$$

In kinematics, there is a relationship between the initial and final velocities of a sphere in the vertical direction as follows:

$$V_{yT} = V_{y0} - gt \tag{S17}$$

Thus we can derive the rising time t of the sphere as:

$$t = \frac{V_{y0}}{g} = \sqrt{\frac{2H}{g}} \tag{S18}$$

Since the motion of the sphere in the horizontal direction can be regarded as uniform velocity motion. Hence, as the motion time t and the horizontal displacement L of the sphere are known, the expression for the initial velocity of the sphere in the horizontal direction V_{x0} is:

$$V_{x0} = \frac{L}{t} = L\sqrt{\frac{g}{2H}} \tag{S19}$$

After obtaining the velocity components of the sphere in the vertical and horizontal directions, respectively, it is easy to determine the initial kinetic energy of the sphere:

$$E_0 = \frac{1}{2}mV_{x0}^2 + \frac{1}{2}mV_{y0}^2 = \frac{1}{4}mg\frac{L^2}{H} + mgH \tag{S20}$$

7. The system trigger source—the Novec 7000 liquid pouch fabrication process

By referencing previous studies by others on liquid pouch fabrication methods,[4–7] we designed a liquid pouch fabrication process as shown in Figure S11. We cut the metallized film to the target size and employed an impulse heat sealer to heat seal three sides of the two layers of film. A pipette was used to inject a measured amount of Novec 7000 from the opening into the liquid pouch, and then the fourth side was sealed using the same procedure to make a completely sealed liquid pouch.

Under the same external heat source stimulation, the expansion capacity of the liquid pouch is positively correlated with the volume of the injected liquid within a certain range. To calculate the Novec 7000 injection volume, we first required the information regarding the volume variation characteristic of the liquid pouch. For the liquid pouches we have fabricated based on rectangular films, assuming that their expanded surfaces are cylindrical, the following geometric relationships can be obtained:[8, 9]

$$L_0 = 2r\beta \tag{S21}$$

$$r\sin\theta = \frac{L}{2} \tag{S22}$$

The geometries of the pouch L_0, r, β, and L are illustrated in Figure S11. Where L_0 is the original width of the liquid pouch when it is not inflated, r is the radius of the surface curvature, β is the central angle of the circular arc, and L is the width of the liquid pouch after inflation. According to Equation (S19) and Equation (S20), by eliminating the radius r, the width of the liquid pouch can be derived as:

$$L(\beta) = L_0\frac{\sin\beta}{\beta} \tag{S23}$$

Therefore, the cross-sectional area A of the liquid pouch after inflation can be calculated. Given the length D of the liquid pouch, the volume of the liquid pouch at any β can be expressed by the following formula:

$$V(\beta) = AD = \frac{L_0^2 D}{2}\left(\frac{\beta - \cos\beta\sin\beta}{\beta^2}\right) \tag{S24}$$

Ideally, the liquid pouch is fully inflated to its maximum volume when Beta = $\pi/2$. The maximum volume of the liquid pouch in this situation is:

$$V_{max} = V(\pi/2) = \frac{L_0^2 D}{\pi} \tag{S25}$$

Combining the gas state equation $PV = nRT$, the volume of Novec 7000 added to the pouch can be determined by the following equation:[4, 7]

$$\begin{aligned} V_l &= \frac{m}{\rho} \\ &= \frac{Mn \times 10^{-3}}{\rho} \\ &= \frac{M}{\rho} \cdot \frac{P}{RT} V_g \times 10^{-3} \\ &= \frac{M}{\rho} \frac{P}{RT} \frac{L_0^2 D}{\pi} \times 10^{-3} \end{aligned} \tag{S26}$$

where m [kg] is the mass of the liquid, M $[\mathrm{g\ mol^{-1}}]$ is the molar mass of the substance, and ρ $[\mathrm{kg\ m^{-3}}]$ is the density of the liquid, and R is the universal gas constant. P and T are the pressure and temperature of the pouch we expect the it to reach.

8. Characterization of Novec 7000 liquid pouches

Through the fabrication process and theory presented in Supplementary Text 7, we fabricated a series of Novec 7000 liquid pouches with the same length (D = 32 mm) and L_0 values of 3 mm, 7 mm, 13 mm, 18 mm, and 25 mm. To test the actuation capability of these pouches under thermal expansion, they were tested on the motorized force tester on the same heating source (a 10 mm × 50 mm polyimide heating film with a maximum power of 1.5 W). Figure S12A illustrates the detailed testing method. The pouch is fixed to the bottom platform of the force tester after being attached to the heating film. Adjust the height of the tester probe so that it is in full contact with the pouch surface, and then set the force reading to zero. After being heated, the pouch tends to expand but its displacement is restricted, so the data acquired by the force tester indicates the expansion capacity of the pouch.

We measured the five different sizes of Novec 7000 liquid pouches we had fabricated individually to obtain force-time data. As shown in Figure S12B, the results of expansion force measurements of the pouch with a 32 mm × 7 mm size under different heating powers. It can be found that the pouch has almost reached the vapor-liquid equilibrium state after 50 seconds of heating and returns to the initial state within 30 seconds after the heating is removed. To better evaluate the actuation capability of the Novec 7000 liquid pouches with various sizes in different scenarios, including the fast response capability and the ultimate maximum output, we extracted the expansion force of the liquid pouches at 15 seconds and 150 seconds under heating and graphed them as Figure S12C and Figure S12D, respectively. The 32 mm × 7 mm pouch demonstrated optimal expansion ability in both scenarios, and the magnitude of the expansion force is sufficient to overcome the $-F_{cr}$ of the VSBBs we tested in the main text. Therefore, we finally chose the 32 mm × 7 mm pouch as the initial trigger source for the series-connected bistable energy storage and transfer (BEST) system in the rest of the experiments.

9. Setup of the series-connected BEST system applications

For the demonstration of the system assisting in the precise delivery of small robotics or critical cargos, we selected a Lego minifigure as the delivery object and printed a base that can dock with the VSBB, ensuring the minifigure can move with the VSBB and eject at the termination point,as shown in Figure S13A. Subsequently, in order to adapt to different application scenarios and obstacle types, we 3D-printed a series of support blocks with different angles to adjust the initial angle of the BEST system. For the obstacle vaulting demonstration, the angle of the support block is 75° and for the vector projecting demonstration, the angle of the support block is 30°.

We handcrafted a glider model made of balsa wood (a lightweight material typically employed in aircraft model crafting), with the specific structure shown in Figure S13B. The final glider model has a wingspan of 368 mm and a weight of 5.07 g. By placing a counterweight on the head of the glider and designing a block at the bottom, we ensured that when it is mounted on the BEST system, the glider model can move with the VSBB without causing any friction with the system frame, thereby ensuring that the energy released by the system is converted into the kinetic energy of the glider.

During the tests, the BEST system was located on a platform 720 mm high. And a PI heating film powered by a DC power supply (heating power: 0.6 W) was employed to heat the Novec 7000 pouch prepared in Supplementary Text 8 as the initial trigger source for the system. Two cameras were used to film the tests, one camera was positioned to record the entire process from system activation to glider gliding from the front, while the other camera was focused on the BEST system to capture the VSBB triggering and the energy transfer process. To visually demonstrate how different VSBB configurations can contribute to varying levels of energy transfer, we conducted three sets of glider launching trials with VSBBs (all 0.6 mm in thickness) featuring different initial inclination angles. Figure S14 presents the launching performance of a series-connected system composed of three VSBBs with initial inclination angles of 14°, 12°, and 12°, which achieved a glide time of 0.55 s and a glide distance of 1150 mm. In the subsequent two sets of experiments, the series-connected system consisting of three VSBBs with initial inclination angles of 14°, 14°, and 12°enabled the glider model to glide for 1220 mm; and the series-connected system composed of three VSBBs with initial inclination angles of 14°enabled the glider model to glide for 1300 mm.

10. Weight dropping impact measurement and acceleration calculation

In order to quantify the energy dissipation capacity of the BEST system, we designed an experiment to measure the impact force when weights fall freely onto the BEST buffer and plain plate as the indicator. Since the probe of the force tester is located at the top position, we designed the test setup as shown in Figure S17. We first 3D printed two flat plates (polylactic acid) with reserved holes. The center of the first plate has a hole for connecting and securing to the probe of the force tester, and there are three holes in the center of the second plate for fixing the BEST buffer. Moreover, the four corners of the two plates have identical holes, which allow the plates to be fixed with each other together as a unit via four M6 threaded rods. Therefore, through the above setup, the impact force after the weight falls can be acquired by the force tester. The sampling rate of the force tester was set to 1 kHz to capture the instantaneous impact process.

During the experiments, the weight (100 grams) was adjusted to the target height and then released, while the force tester acquired time domain data before and after the impact. Since the mass m of the weight is known, the time variation of the impact acceleration can be calculated using $F = ma$. Further, we divided the acceleration results by 9.8 to obtain results based on the acceleration of gravity g as the basic unit. The experiments were conducted with the buffer and without the buffer (in this case the weight was dropped directly onto the plain plate), and three tests were carried out at each height. Apart from verifying the outstanding energy dissipation capability of the BEST buffer through impact acceleration results in the time domain, we subsequently applied the short-time Fourier transform method to obtain the spectrogram, which explained the excellent damping property of the BEST buffer from an energy perspective. The data processing was performed using the scipy.signal package in Python.

Supplementary Figures

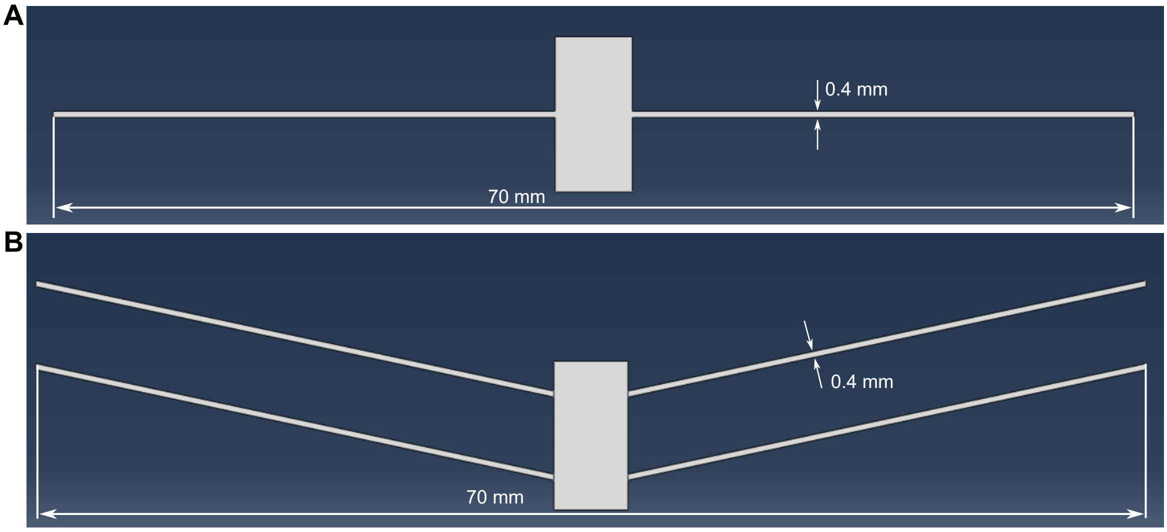

Figure S1. Two-dimensional models for for force-displacement simulations. A). Two-dimensional model of DCBB. B). Two-dimensional model of VSBB.

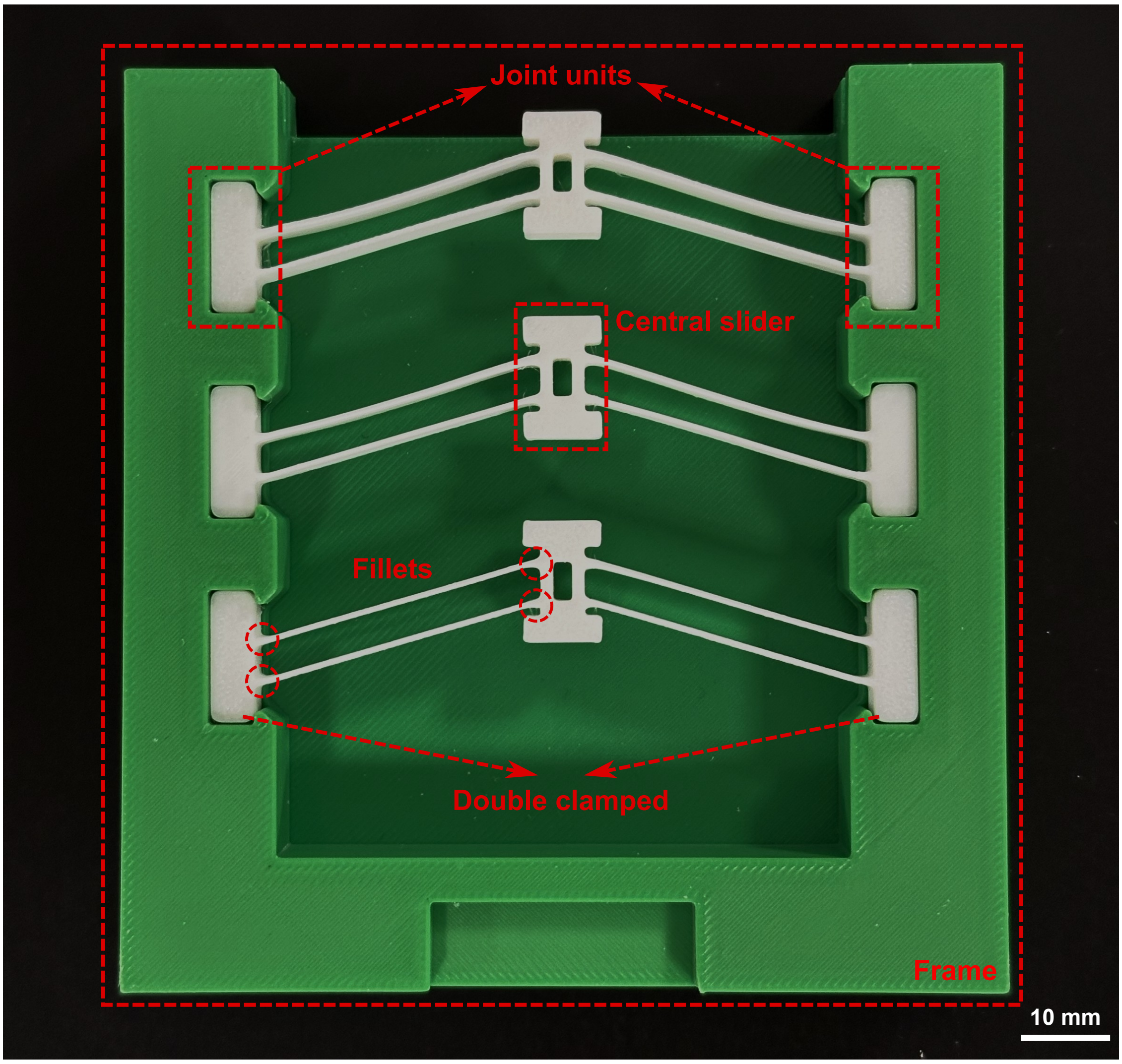

Figure S2. Details of individual modules of the BEST system.

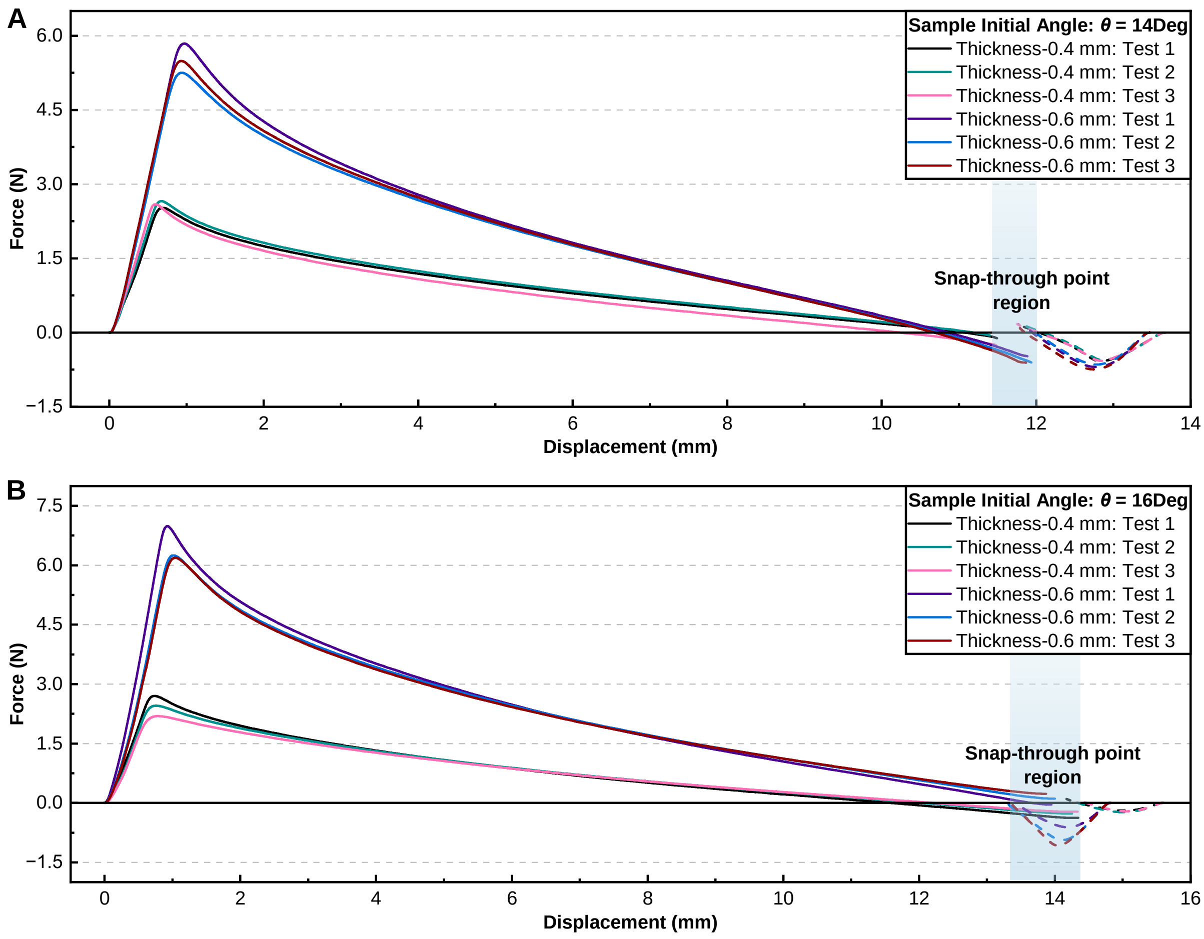

Figure S3. Force-displacement test results of VSBBs. A). Results for the initial inclination angle of 14°. B). Results for the initial inclination angle of 16°. The dashed lines indicate testing performed from SP2.

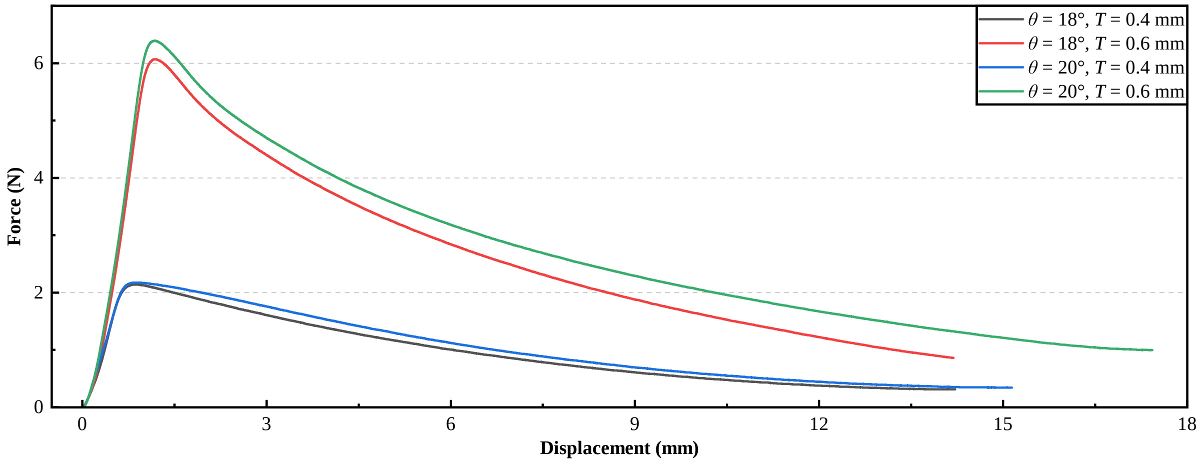

Figure S4. Force-displacement test results of VSBBs with initial inclination angles of 18° and 20° (no snap-through occurred).

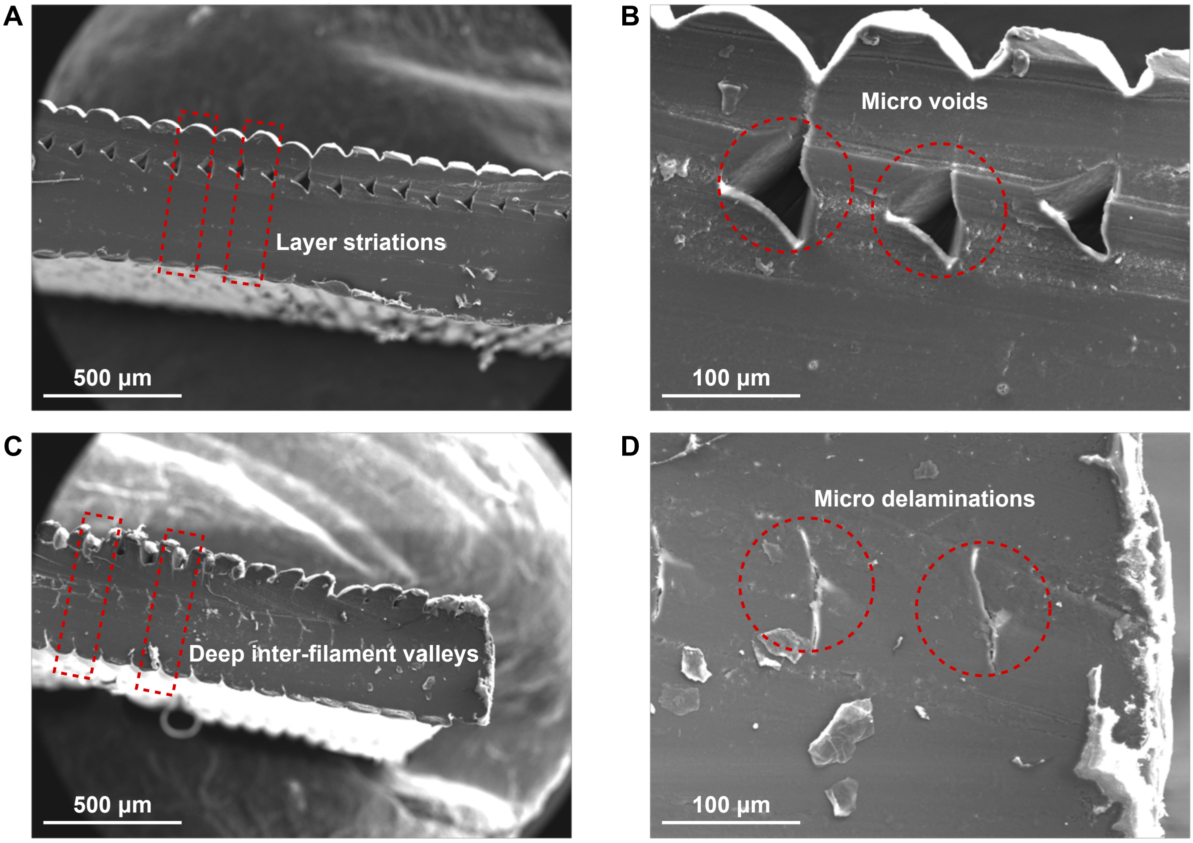

Figure S5. Cross-sectional micrograph of VSBBs with a thickness of 0.4 mm. A). Typical imperfection 1: Layer striations. B). Typical imperfection 2: Micro voids. C). Typical imperfection 3: Deep inter-filament valleys. D). Typical imperfection 4: Micro delaminations.

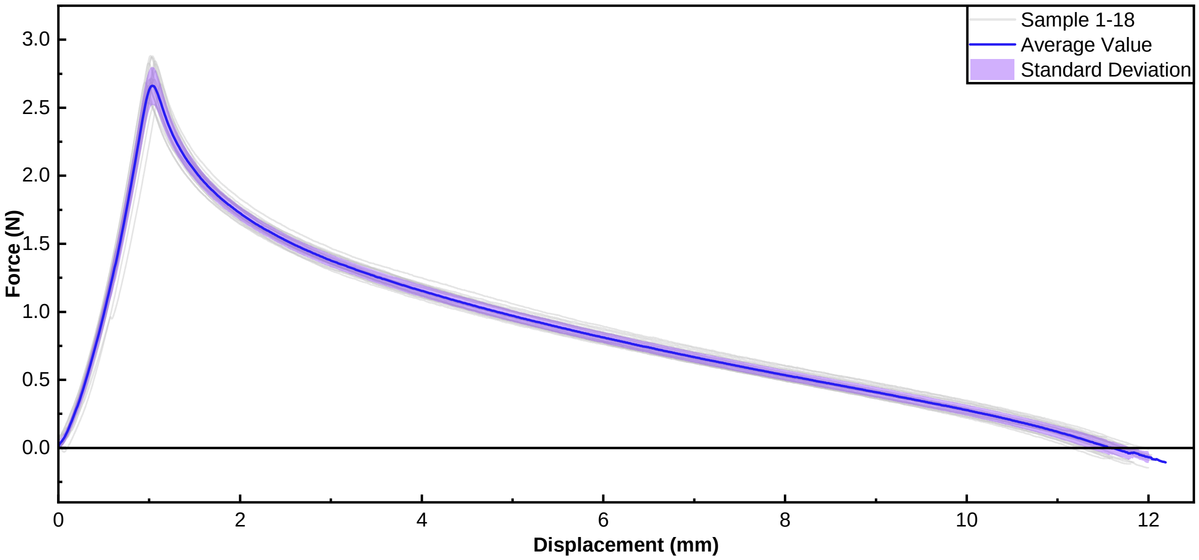

Figure S6. Repeatability measurement results of the force-displacement curve for VSBB with $\theta = 14°$ and $T = 0.4$ mm.

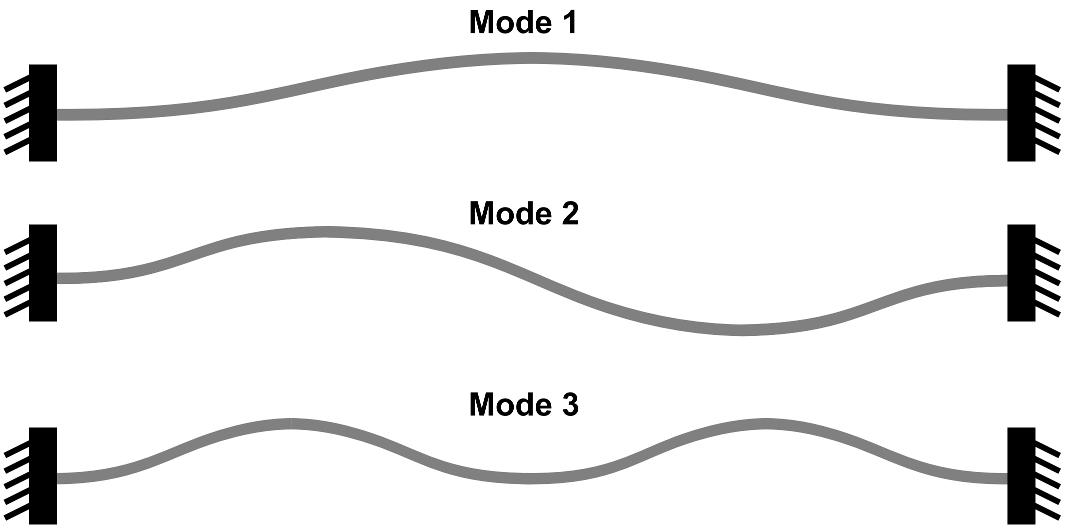

Figure S7. The first three buckling modes for a DCBB.

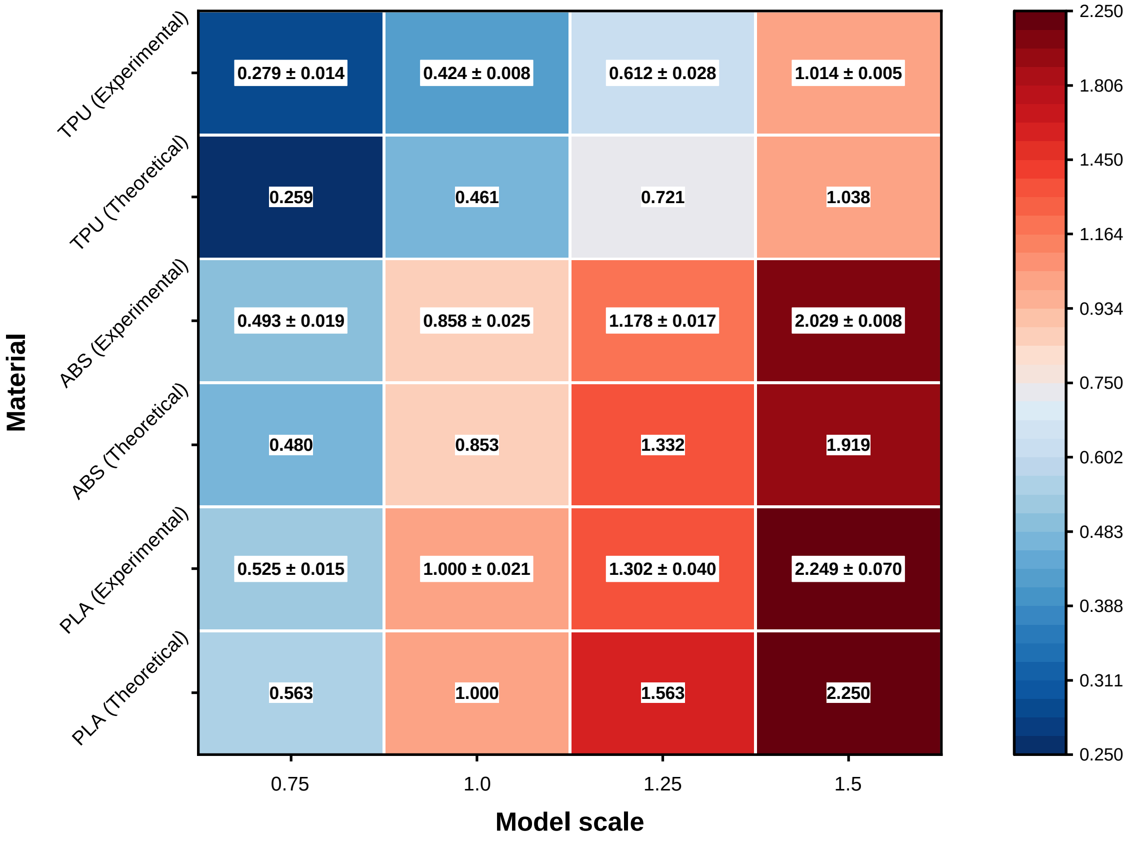

Figure S8. Normalized heatmap of critical buckling load F_{cr} measurement results for VSBB across multiple materials and scales.

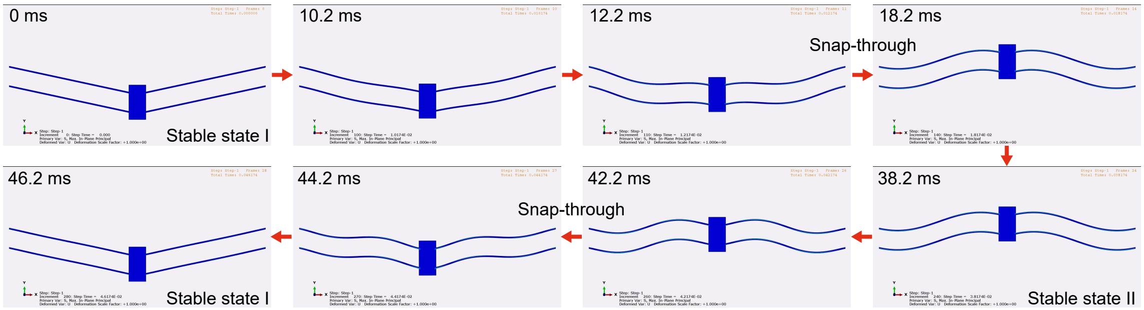

Figure S9. The velocity variation process of VSBB with initial inclination angle of 12° simulated by the implicit dynamic analysis method.

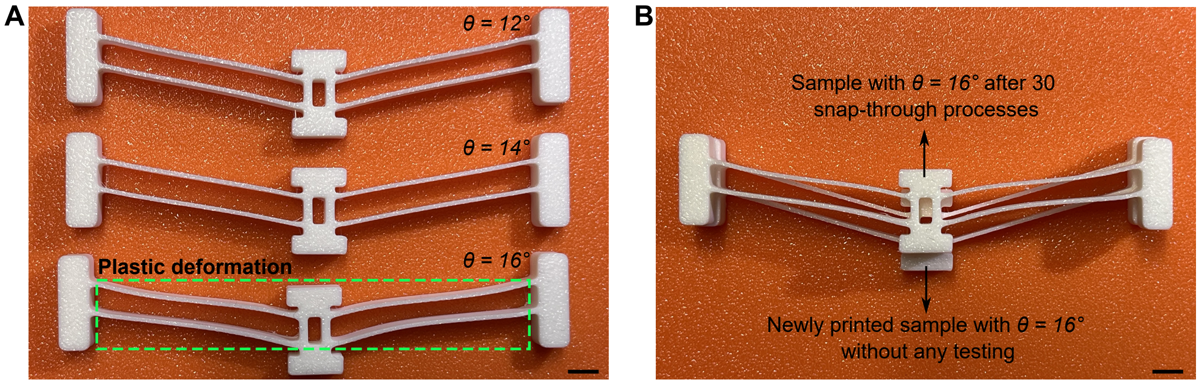

Figure S10. Plastic deformation of the 3D-printed VSBB. A). Comparison of the shapes of VSBBs with different initial inclination angles after 30 snap-through processes. B). Comparison of the shape of a new VSBB with an initial inclination angle of 16° and a VSBB with an initial inclination angle of 16° that has been treated with 30 snap-through processes. scale bar, 5 mm.

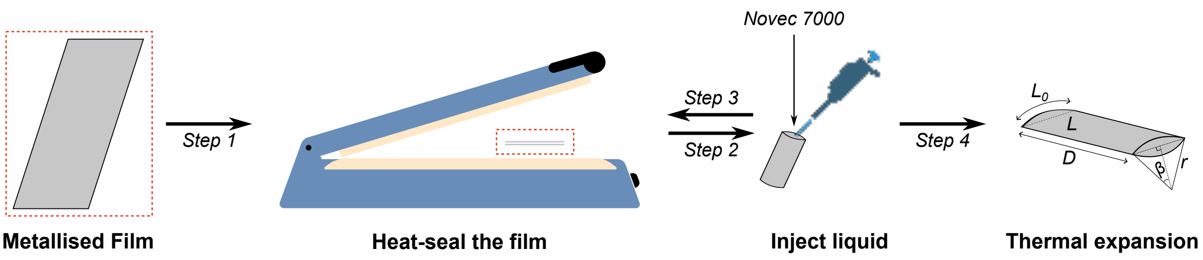

Figure S11. The process of fabricating Novec 7000 liquid pouch through hot pressing method.

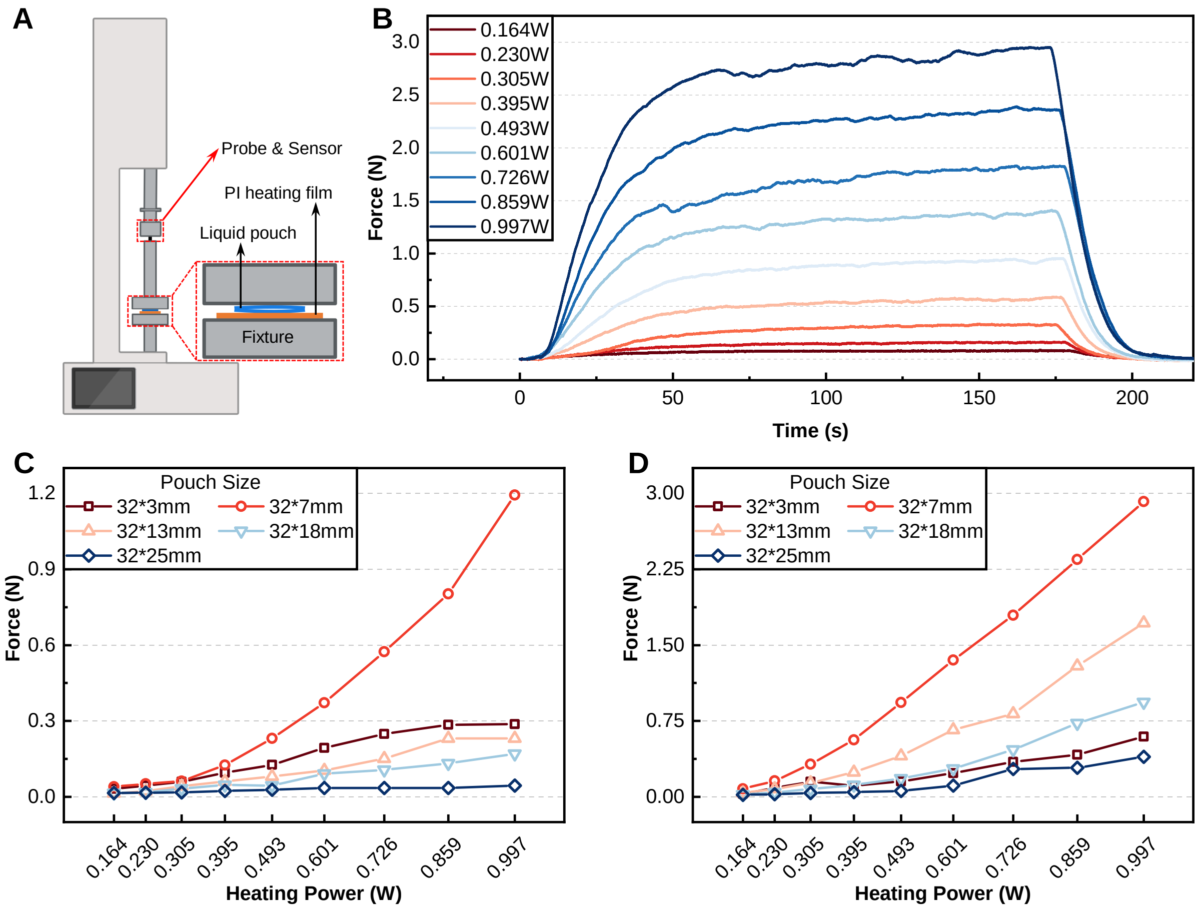

Figure S12. Characterization of Novec 7000 liquid pouches. A). Schematic diagram of the test system for measuring the expansion capacity of liquid pouches. B). Force measurement results of a liquid pouch with an initial size of 32 × 7 mm under different heating powers. C). Force measurement results for liquid pouches with different initial sizes heated at different heating powers for 15 seconds. C). Force measurement results for liquid pouches with different initial sizes heated at different heating powers for 150 seconds.

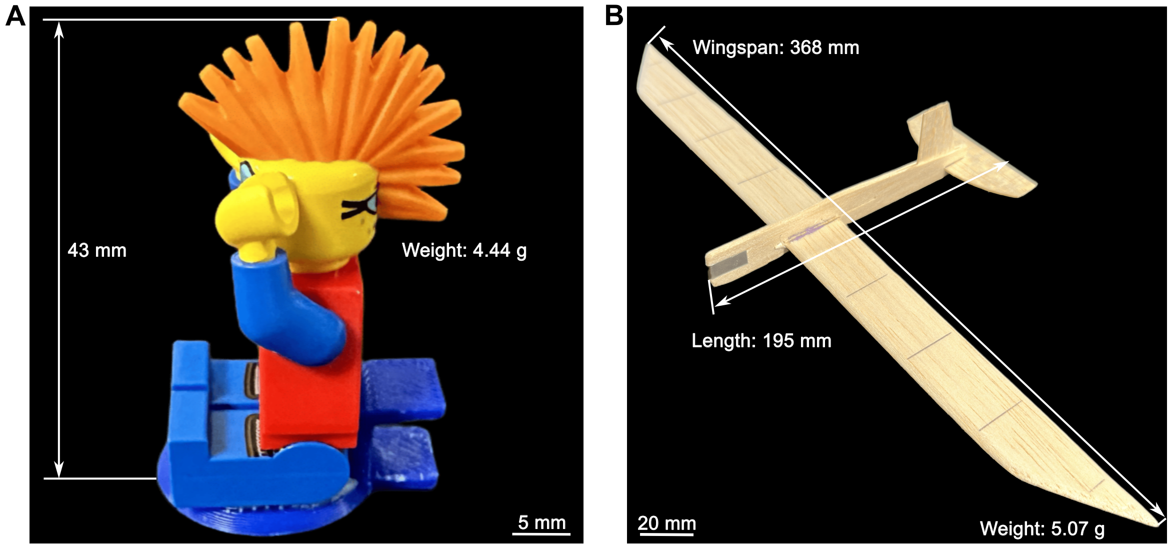

Figure S13. The object details for the series-connected BEST system applications. A). The mini robot information for precise delivery. B). The handcrafted glider information.

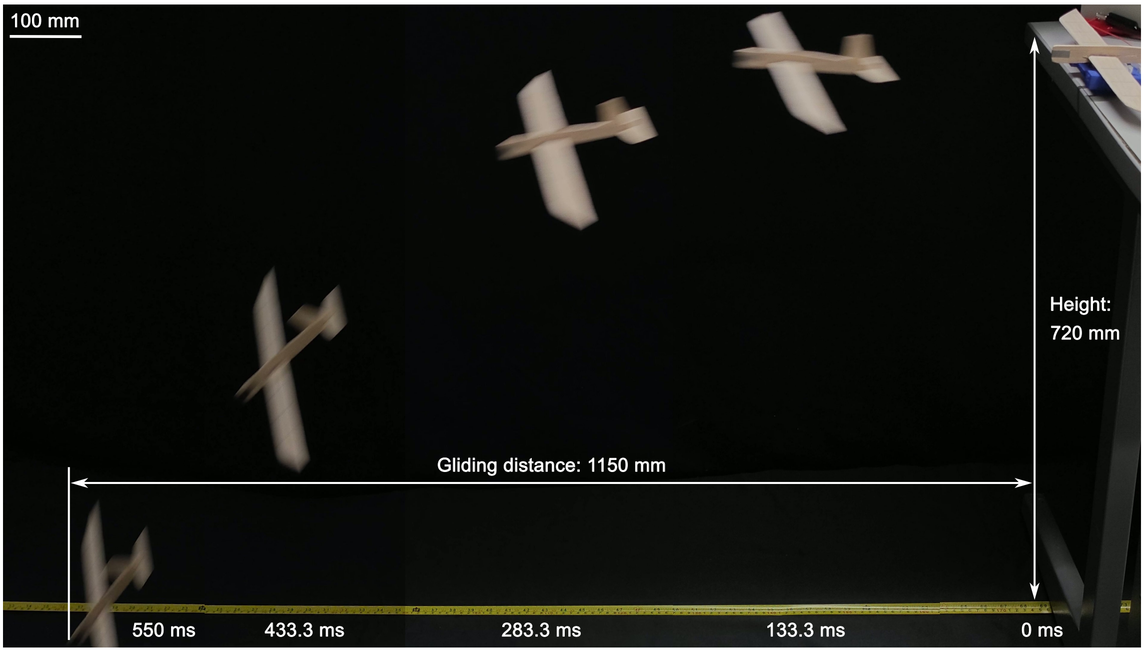

Figure S14. Glider launching demonstration result (VSBB initial inclination angle combination: 12° , 12° , and 14°).

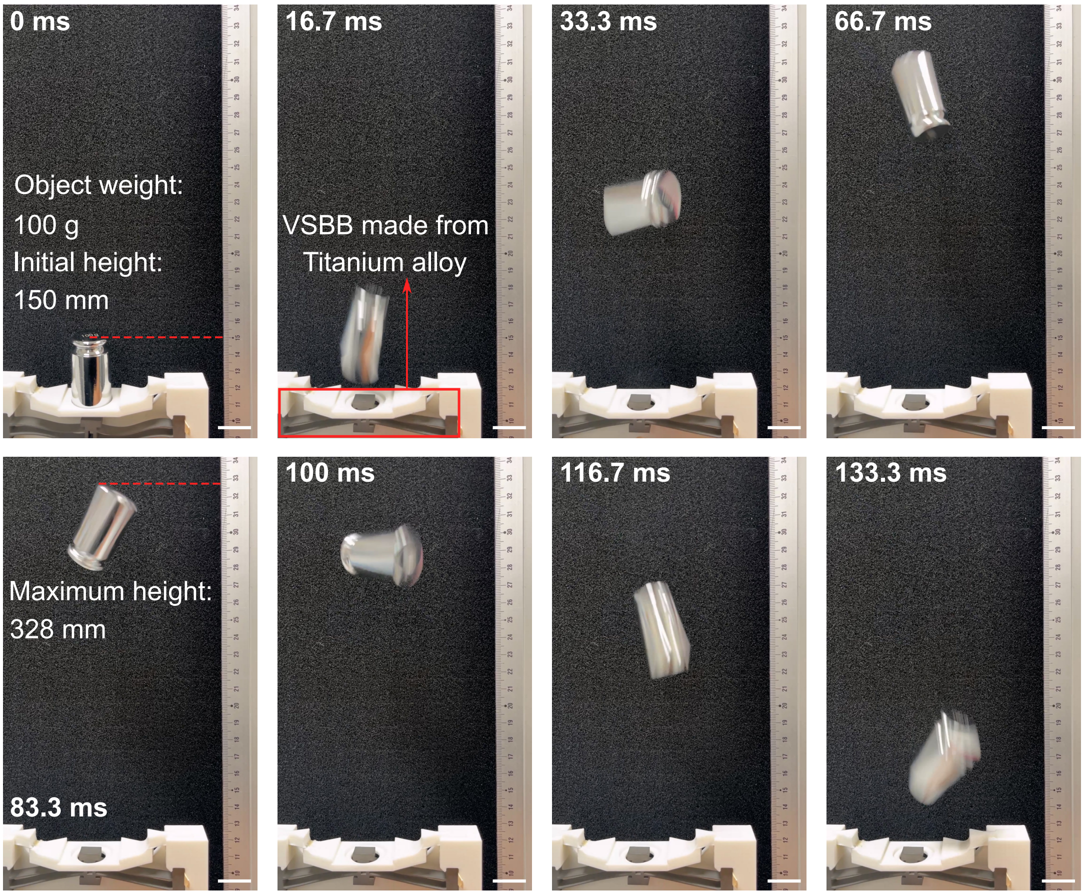

Figure S15. The demonstration performance of energy release from the VSBB made of titanium alloy (VSBB model with θ = 14° and T = 0.6 mm, scaled up by 1.25 times). Scale bar, 10 mm.

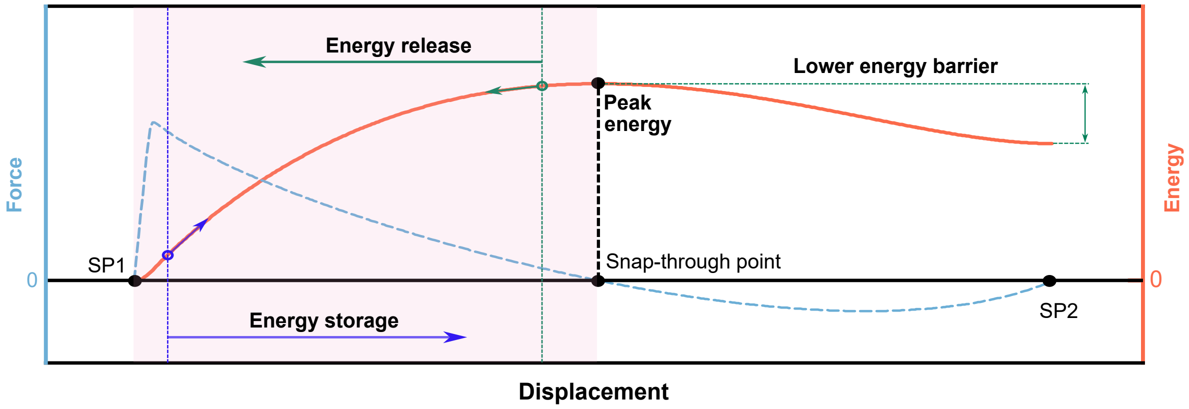

Figure S16. Correspondence between energy landscape and applications of V-shaped bistable structure.

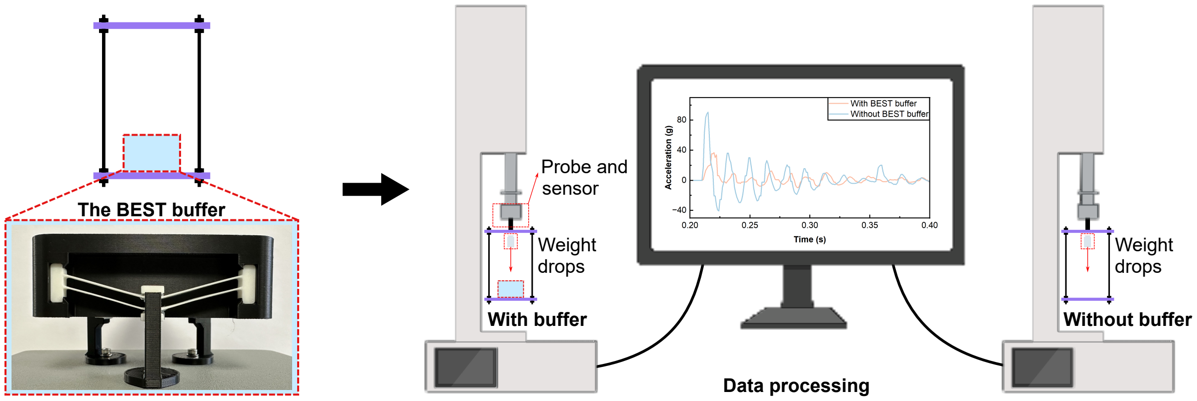

Figure S17. Experiment setup for measuring the impact of weight dropping.

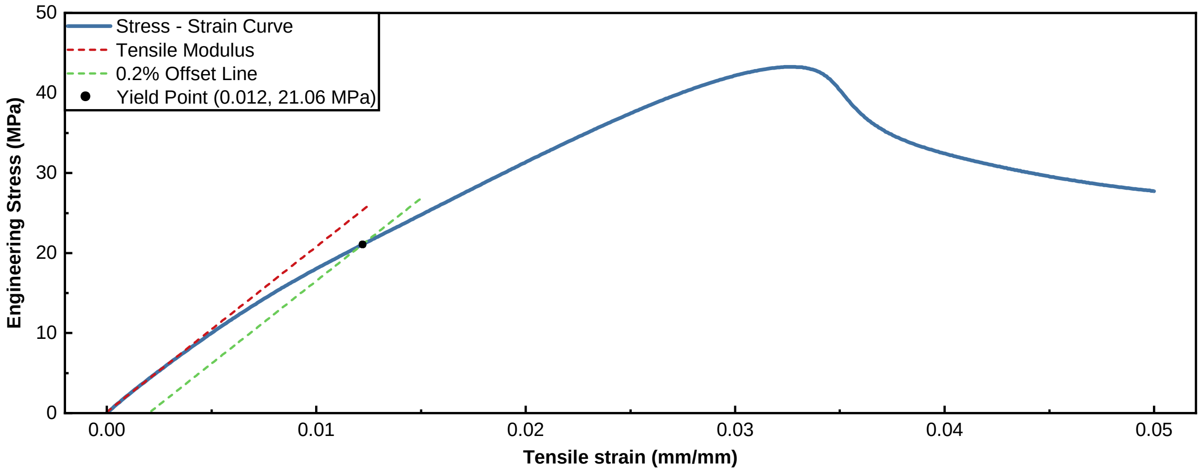

Figure S18. Stress-strain curve during the tensile test of the specimen based on ASTM D638-14 and the material properties determination method.

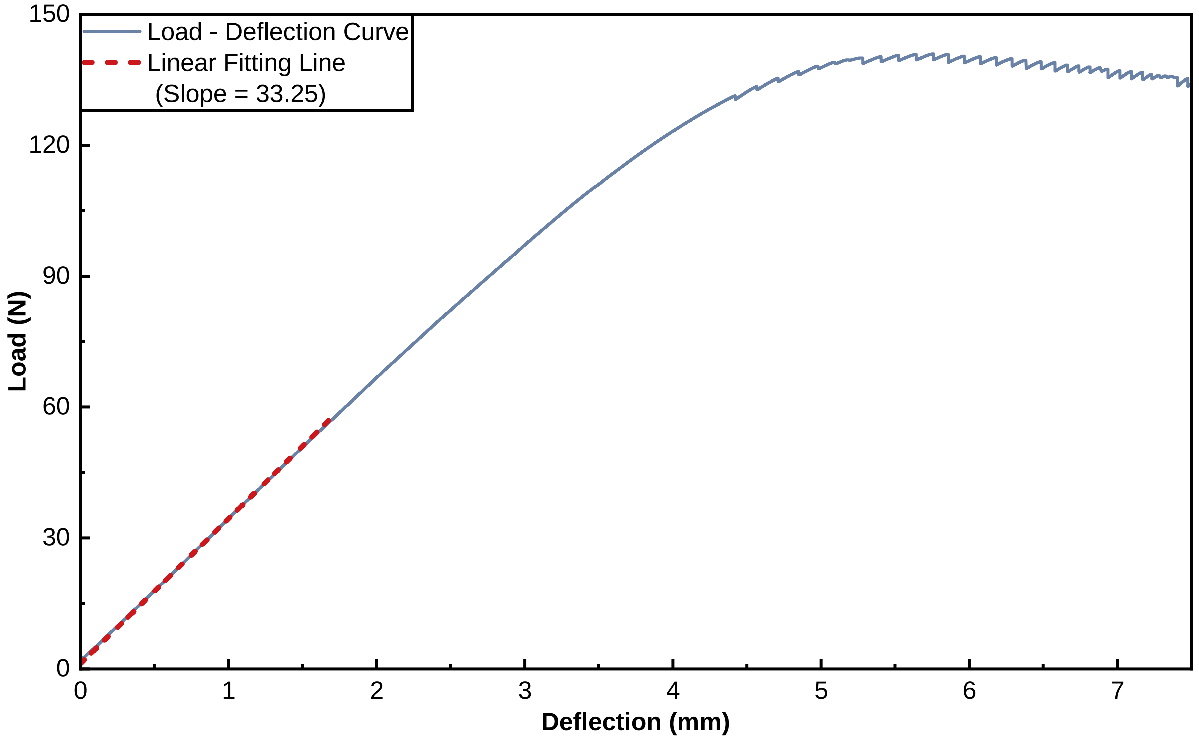

Figure S19. Load-deflection curve during the three-point bending test of the specimen based on ASTM D790-17 and the material properties determination method.

Supplementary Table

Table S1. Summary of features of the recent robotic/actuator systems achieving autonomous motion using bistable systems. (BL: Body length)

Structural form	Main material/ mechanism	Actuation method	Size	Mass	Velocity/Displacement	Ref.
Y-shaped flexure hinge	Shape-memory alloy spring	Joule heating	30 mm (width)<br>58 mm (length)<br>58 mm (height)	9.7 g	Jump height: 140.6 mm (2.5 BL)<br>Jump distance: 230 mm (4 BL)	[10]
Soft body<br>+<br>Actuator	Twisted and-coiled actuator Spring	Joule heating	—	—	Jump height: 70 mm (> 5 BL)	[11]
Soft body<br>+<br>Spring	Ecoflex<br>Spring	Pneumatic	7 cm (length)<br>6 cm (wide)	45 g	Linear locomotion speed:<br>187.5 mm/s (2.68 BL/s)	[12]
Bistable beam	Spring steel	Pneumatic	28 mm (length)	2.4 g	Jump height: 12.7 BL<br>Jump distance: 20 BL	[13]
VSBB	PLA	Liquid pouch	70 mm (width)<br>0.6 mm (beam thickness)<br>6 mm (height)	< 2 g<br>(Single VSBB)	Jump height: 359 mm (> 35 BL)<br>Speed: 2655 mm/s (> 265 BL/s)<br>Project object: Steel sphere<br>(10 mm in diameter, 4 g in mass)	**This work**

Table S2. Results of the average input and transferred energy for VSBBs with different geometric parameters. The input energy was calculated as the area enclosed by the D_{SR} segment curve and the X-axis.

Sample geometries	Energy input (mJ)	Energy transferred (mJ)	Ratio
**12°-0.4 mm**	0.284	3.243	11.4
**12°-0.6 mm**	0.638	9.326	14.6
**14°-0.4 mm**	0.479	3.617	7.6
**14°-0.6 mm**	0.633	10.125	16.0
**16°-0.4 mm**	0.578	5.576	9.6
**16°-0.6 mm**	0.728	11.472	15.8

Table S3. Material parameters of 3D-printed PLA material in the primary deformation direction.

Elastic		Yield strain	Plastic	
**Tensile modulus (MPa)**	**Bending modulus (MPa)**		**Yield stress (MPa)**	**Plastic strain (mm/mm)**
2051 ± 92	2299 ± 23	1.19%	20.62	0
			21.60	0.0020
			30.27	0.0040
			37.66	0.0061
			43.66	0.0094

Legends for Supplementary Movies

Movie S1. The BEST system application in vector projecting.
The series-connected BEST system is linked with the delivery object, and the target destination is a pipe located approximately 460 mm ahead and 30°upward. In order to achieve accurate vector projection, an initial tilt angle is provided to the system by using a 30° support block. To supply the delivery object with sufficient initial kinetic energy, three VSBBs with initial inclination angle of 14° and beam thickness of 0.6 mm are selected to form the system. The Novec 7000 liquid pouch is heated by a PI heating film at a power of 0.6 W. After the system is triggered, the object successfully enters the pipe after approximately 300 ms.

Movie S2. The BEST system application in obstacle vaulting.
The series-connected BEST system is linked with the delivery object and arranged at a distance of 120 mm from the obstacle via a support block with an angle of 75° . To guarantee the object can obtain enough energy to overcome the 384 mm height barrier, three VSBBs with initial inclination angle of 14° and beam thickness of 0.6 mm are selected to form the system. The Novec 7000 liquid pouch is heated by a PI heating film at a power of 0.6 W. The system is triggered approximately 20 seconds after heating started, and then the object takes around 530 ms to cross the obstacle and reach its destination.

Movie S3. The BEST system application in programmable precision ingress I.
The series-connected BEST system is linked with the delivery object and deployed at a height of 320 mm. The target area is approximately 680 mm ahead. Three VSBBs with initial inclination angle of 14°, 14°, 12°and beam thickness of 0.6 mm are selected to form the system. The Novec 7000 liquid pouch is heated by a PI heating film at a power of 0.6 W. The system is triggered approximately 30 seconds after heating started, and then the object takes around 350 ms to reach its destination.

Movie S4. The BEST system application in programmable precision ingress II.
The series-connected BEST system is linked with the delivery object and deployed at a height of 320 mm. The target area is approximately 800 mm ahead. Since the target area for this mission is farther away than programmable precision ingress I, the object needs to acquire greater initial kinetic energy. Therefore, three VSBBs with initial inclination angle of 14°and beam thickness of 0.6 mm are selected to form the system. The Novec 7000 liquid pouch is heated by a PI heating film at a power of 0.6 W. The system is triggered approximately 28 seconds after heating started, and then the object takes around 350 ms to reach its destination.

Movie S5. The BEST system application in programmable small-scale glider launching I.
The series-connected BEST system is linked with the glider model and deployed at a height of about 720 mm. Three VSBBs with initial inclination angle of 14°, 12°, 12°and beam thickness of 0.6 mm are selected to form the system. The Novec 7000 liquid pouch is heated by a PI heating film at a power of 0.6 W. The system is triggered approximately 33 seconds after heating starts, then the glider is released to begin gliding and lands at a distance of 1150 mm from the starting point.

Movie S6. The BEST system application in programmable small-scale glider launching II.
The series-connected BEST system is linked with the glider model and deployed at a height of about 720 mm. Three VSBBs with initial inclination angle of 14°, 14°, 12°and beam thickness of 0.6 mm are selected to form the system. The Novec 7000 liquid pouch is heated by a PI heating film at a power of 0.6 W. The system is triggered approximately 33 seconds after heating started, then the glider is released to begin gliding and lands at a distance of 1220 mm from the starting point.

Movie S7. The BEST system application in programmable small-scale glider launching II.
The series-connected BEST system is linked with the glider model and deployed at a height of about 720 mm. Three VSBBs with initial inclination angle of 14°and beam thickness of 0.6 mm are selected to form the system. The Novec 7000 liquid pouch is heated by a PI heating film at a power of 0.6 W. The system is triggered approximately 33 seconds after heating started, then the glider is released to begin gliding and lands at a distance of 1300 mm from the starting point.

Movie S8. Concept demonstration of the BEST system as a vascular stent.
Six VSBBs with initial inclination angle of 14°and beam thickness of 0.6 mm are installed in a hexagonal cross-section framework to form a parallel-connected BEST system. A balloon connected to an external pneumatic supply is placed in the center of the system. The circular ring formed by origami is utilized to simulate elastic blood vessel. After the system is triggered, the diameter of the ring rapidly expands from 63 mm to 75 mm within 50 ms.

Movie S9. Demonstration of magnetic effect as a trigger source.
We select two N42 neodymium magnets with a diameter of 8 mm and a thickness of 2 mm. One is connected to the VSBB, and the other is used to generate a repulsive force between the two with the same polarity. First, VSBB with an initial inclination angle of 12°and a beam thickness

of 0.4 mm is tested. The repulsive force generated by the two magnets as they approach each other enables the VSBB to snap-through. Second, VSBB with an initial inclination angle of 14°and a beam thickness of 0.6 mm is also successful snap-through. Finally, Two VSBBs with initial inclination angle of 14°and beam thickness of 0.6 mm are connected serially, which is triggered smoothly.

Movie S10. Demonstration of energy dissipation property: raw egg falling test.
First, an egg is placed on a 3D-printed plate and then released from a height of approximately 300 mm above the bottom plate. As a result, the egg breaks upon impact after landing. In contrast, we fix the buffer composed of three VSBBs with an initial inclination angle of 14°and beam thickness of 0.6 mm to the bottom plate with screws. Then, an egg is released from a height of approximately 350 mm above the top surface of the buffer. As a result the egg remains intact after landing.

References

1. Timoshenko, S. P. & Gere, J. M. *Theory of elastic stability* (Courier Corporation, 2012).
2. Vangbo, M. An analytical analysis of a compressed bistable buckled beam. *Sensors and Actuators A: Physical* **69,** 212–216 (1998).
3. Qiu, J. A curved-beam bistable mechanism. *Journal of microelectromechanical systems* **13,** 137–146 (2004).
4. Narumi, K. *et al.* Liquid pouch motors: printable planar actuators driven by liquid-to-gas phase change for shape-changing interfaces. *IEEE Robotics and Automation Letters* **5,** 3915–3922 (2020).
5. Çelebi, M. F. *et al. Textile-based thermally driven actuators for soft robotic mechanotherapy applications* in *2022 8th International Conference on Control, Decision and Information Technologies (CoDIT)* **1** (2022), 1303–1308.
6. Yamaguchi, S., Hiraki, T., Ishizuka, H. & Miki, N. Handshake Feedback in a Haptic Glove Using Pouch Actuators. *Actuators* **12.** ISSN: 2076-0825. https://www.mdpi.com/2076-0825/12/2/51 (2023).
7. Exley, T. *et al. Agonist-Antagonist Pouch Motors: Bidirectional Soft Actuators Enhanced by Thermally Responsive Peltier Elements* in *2024 IEEE/RSJ International Conference on Intelligent Robots and Systems (IROS)* (2024), 9214–9220.
8. Niiyama, R., Rus, D. & Kim, S. *Pouch motors: Printable/inflatable soft actuators for robotics* in *2014 IEEE International Conference on Robotics and Automation (ICRA)* (2014), 6332–6337.
9. Niiyama, R. *et al.* Pouch motors: Printable soft actuators integrated with computational design. *Soft Robotics* **2,** 59–70 (2015).
10. Zhakypov, Z., Mori, K., Hosoda, K. & Paik, J. Designing minimal and scalable insect-inspired multi-locomotion millirobots. *Nature* **571,** 381–386 (2019).
11. Sun, J., Tighe, B. & Zhao, J. *Tuning the energy landscape of soft robots for fast and strong motion* in *2020 IEEE International Conference on Robotics and Automation (ICRA)* (2020), 10082–10088.
12. Tang, Y. *et al.* Leveraging elastic instabilities for amplified performance: Spine-inspired high-speed and high-force soft robots. *Science advances* **6,** eaaz6912 (2020).
13. Guo, Q. *et al.* Bistable Insect-Scale Jumpers with Tunable Energy Barriers for Multimodal Locomotion. *Advanced Science* **11,** 2404404 (2024).
